# Evaluating the Influence of the Client Behavior in Cloud Computing

**DOI:** 10.1371/journal.pone.0158291

**Published:** 2016-07-21

**Authors:** Mário Henrique Souza Pardo, Adriana Molina Centurion, Paulo Sérgio Franco Eustáquio, Regina Helena Carlucci Santana, Sarita Mazzini Bruschi, Marcos José Santana

**Affiliations:** Institute of Mathematical and Computer Sciences, University of São Paulo, São Carlos, Brazil; Tianjin University of Technology, CHINA

## Abstract

This paper proposes a novel approach for the implementation of simulation scenarios, providing a client entity for cloud computing systems. The client entity allows the creation of scenarios in which the client behavior has an influence on the simulation, making the results more realistic. The proposed client entity is based on several characteristics that affect the performance of a cloud computing system, including different modes of submission and their behavior when the waiting time between requests (think time) is considered. The proposed characterization of the client enables the sending of either individual requests or group of Web services to scenarios where the workload takes the form of bursts. The client entity is included in the CloudSim, a framework for modelling and simulation of cloud computing. Experimental results show the influence of the client behavior on the performance of the services executed in a cloud computing system.

## 1. Introduction

Cloud computing has brought about the concept of computing as a utility, making software, platform and infrastructure to be sold as services. Companies and developers that have business on the Internet do not need to worry about computational infrastructure to bring their projects into operation. The infrastructure can be leased in a cloud provider, which handle different demands of workloads and prevent resources from remaining idle or overloaded [1].

One of the most considered features of cloud computing systems is related to the way resources are allocated. In cloud providers, the resources allocated to fulfill the demands of workload imposed by the users are provisioned dynamically, and may increase or decrease, as required. Service Level Agreements (SLAs) must be defined for the regulation of all the details of computing resources allocation as well as the pricing of the service provisioned by the cloud [2, 3].

Providers of cloud computing have a robust infrastructure that consists of servers, switches, routers, air conditioning devices, structured cabling, power supplies, energy, and other. Cloud computing systems involve different types of devices and are large-scale systems, which makes the prototyping of this magnitude quite difficult. Researchers worldwide have used simulation to create different ways of evaluating the behavior of the systems and validating strategies for task scheduling, cost models, scaling with energy efficiency, etc. [3, 4, 5].

CloudSim [6] is a tool for modeling and simulation of cloud computing environments, with focus on strategies for CPU scheduling, allocation of virtual machines, optimization of energy consumption, among other. Its original version did not address the client´s point of view and this consequently leads to a missing entity in simulation programs. CloudSim concentrates on virtually building the perspective of the provider side. The client is depicted by the Broker, which is an entity of the infrastructure simulated. In most of the studies that use the standard CloudSim API (Application Programming Interface), the workload is submitted from the Broker entity. This behavior does not allow observing the network latency before the arrival of the request to the Broker, i.e. the network latency between client and Broker. Additionally, all requests are submitted to the cloud at the beginning of the simulation, at the same instant of time. During the rest of the simulation, new requests do not arrive at the system. This behavior is not compatible with the reality of computational cloud computing systems because the workload can be unpredictable and highly variable, mainly in those that are open to the public access.

This study complements the CloudSim simulator, as it brings the client entity to the simulation. Therefore, scenarios in which customers influence the simulation can be created, allowing more realistic environments. As an example, it is possible to have scenarios considering the presence of several concurrent customers in different geographic points. These customers can submit workloads with different characteristics, modeled with or without bursts at the requests arrival process. Thus, the presence of the client entity in the simulator enables companies and people to simulate cloud computing providers before hiring one.

The goals of this study include the presentation of the client entity and the illustration of the different forms of submission of requests from clients to the cloud provider. The influence of the workload characteristics imposed by clients is also addressed.

The remainder of this paper is organized as follows: Section 2 discusses the related work; Section 3 introduces the architecture of the CloudSim framework for modeling and simulation cloud computing systems; Section 4 presents the extension for the CloudSim Architecture; Section 5 explains the architecture components for the new proposed client entity approach; Section 6 reports on the design of the simulation experiments; Section 7 discusses the experimental results; Section 8 addresses the conclusions of the work; finally, Section 9 suggests topics for future work.

## 2. Related Work

Several studies in the literature have dealt with the modeling and simulation of cloud computing environments using CloudSim framework [7, 8, 9]. Perret et al. [10] propose a task scheduler for cloud computing systems which handles jobs with execution deadlines. The proposed algorithm named as Cloud Least Laxity First (CLLF) is compared with two task scheduling algorithms included in the original CloudSim called Time Shared and Space Shared. All tasks (called cloudlets in CloudSim) are sent at the beginning of the experiment and ordered in the Broker, from most urgent to least urgent. The authors claim to have performance gains in reducing the number of nodes allocated to the workload and preservation of the execution time required by tasks deadlines.

Kathiravelu and Veiga [11] present an extension to CloudSim called Cloud2Sim. They propose to transform the CloudSim in a parallel and distributed simulator using a computer cluster infrastructure to run the simulations. In this work, the Cloud2Sim architecture was developed by adding some additional layers over the standard of CloudSim architecture. With the features added by new layers, it becomes possible to model cloud computing scenarios with more elements or simulation objects (data centers, hosts, virtual machines, tasks, etc.). Thus, by employing the distributed computer environment cluster, it becomes possible to perform complex simulation programs whose number of objects is large. With the use of some additional technologies such as Hazelcast, Infinispan and Hibernate Search, the new extension of CloudSim adds functions that allow performing parallel and distributed execution of scenarios of cloud computing simulation.

Sá et al. [12] propose another extension to CloudSim. In this work, it is presented a visual tool named CloudReports, which improves the process of reporting the simulation results. The project, proposed as open-source software, provides a simplified API that allows the creation of new features for reports. According to the authors, the CloudReports tool can be integrated with CloudSim API to obtain the execution results and to generate the report layouts. The paper experiments were composed of cloud computing simulations with focus on efficient energy consumption using CloudReports coupled to simulation programs in order to generate theoutput information report. The results show the tool's effectiveness in presenting data and information in a more efficient mode for analysis.

The studies presented in [5] and [13] focus on the use of CloudSim to evaluate cloud computing and green computing. Beloglazov et al. [5] propose an architecture that provides efficient energy consumption in cloud environments; it uses policies and scheduling algorithms to reduce energy consumption while maintaining the service level agreements (SLAs) contracted. In [13], CloudSim is customized to provide data on energy efficiency, such as consumption in KWh, percentage of broken contracts, percentage of CPU usage, among others.

Goutam et al. [14] present techniques for dynamic load balancing for cloud computing environments. The proposed implementation called RAM Broker decides, in a proactive manner, in which cloud resource the workload should be allocated. The proposed approach in this work makes use of CloudSim and considers fault tolerance in their implementation. The proactive mechanism RAM Broker manages the virtual machines allocating incoming workloads and creating new VM instances when needed. According to the authors, the experimental results of the simulation showed the proficiency of RAM Broker in getting performance gains and the use of cloud computing resources.

In general, most of the current work found in the literature considers the approach adopted by CloudSim, i.e., the focus is on the functions of the cloud provider, abstracting the vision of the client. Fittkau et al. [15] report on an experience to provide a perspective of the client in the simulations, introducing enhancements in CloudSim. It is possible to compare different cloud computing providers and select the best features for the client requirements. The paper presents a comparison between the simulation and a real infrastructure (based on Amazon's EC2 service), concluding that the simulation faithfully reproduces the real environment. However, two gaps observed refer to the absence of a simulation entity that truly represents the behavior of clients, i.e., how they submit their requests in the time, and the absence of information on response variables associated with each client.

Some of the gaps observed in the studies presented in this section are filled with the work presented in this paper. In this work, the simulation environment includes the client entity, the client’s behavior, including the different ways to submit the requests, with a time interval between the submissions of two successive requests from the same client, and the workload model considering burstiness phenomenon. This abstraction can emulate different clients that hypothetically run several different types of applications, such as uploading or downloading files, requesting a web service, querying the contents of websites, among many other possibilities.

## 3. CloudSim Architecture

CloudSim is a robust framework that allows the creation of simulations of cloud environments through the extension of existing classes in its Application Programming Interface (API) [6, 7]. Both the behavior of systems in Cloud Computing (task scheduling, allocation policies for virtual machines, scheduling policies) and the system infrastructure (data centers, virtual machines, hosts, processors, bandwidth network, storage, and others) can be modelled. CloudSim adopts a modular multilayer architecture [6], namely User Code, CloudSim and Core Simulation Engine, to manage its components separately.

The lower layer of the CloudSim architecture, CloudSim Core Simulation Engine, is responsible for the events and queue managers used by the simulator. This layer handles the interaction between CloudSim entities and components. All components in CloudSim communicate through message passing operations.

The CloudSim layer supports the modeling and simulation of cloud computing in virtualized environments, including dedicated management interfaces for virtual machines, memory, storage, and bandwidth. This layer is also used to manage the instantiation and execution of several simulation components (virtual machines, hosts, data centers and other). The provisioning of the virtual machines, managing of the applications (or cloudlets) execution and monitoring of the system status are also handled in the CloudSim layer. A cloud provider who wants to evaluate, for example, different policies in allocating its hosts to Virtual Machines (VMs), would need to implement his strategies in this layer, extending the core VM provisioning functionality. This layer also has a module that contains information about the network topology. In the initial releases, until CloudSim 2.1, the simulator had no support for simulation of network entities such as routers or switches; instead, the latency of a message from a source entity to a target entity was used. Since Cloudsim version 3.0, an extension was added for network topologies manipulation within data centers, named NetworkCloudSim, including classes aimed to model hubs and switches [16]. New classes to support applications with communicating elements or tasks such as Message Passing Interface (MPI) and workflows were also implemented. The CloudSim simulator, even in its latest version (3.0.3), does not have an entity that represents the Cloud Computing client. Despite the new network characterization and message passing resources of the CloudSim, it is not possible to represent a client request considering its source and latency’s aspects applied to this important element that is part of the Computer Architecture Cloud.

The User Code layer, located on the top of the layers, shows the simulation scenario settings related to specifications of the hosts (servers), applications, virtual machines, and Broker scheduling policies (Round Robin). The Broker entity acts in two different ways, i.e., as a mediator between SaaS and the cloud provider and as a representation of the client.

There is no client entity in the studies that use the standard CloudSim API. Instead, a single set of requests is sent at the beginning of the simulation, through the submitCloudletList method of the DatacenterBroker component and then to the VMs in the entity Datacenter for execution. In the standard CloudSim implementation, there is a scheduling algorithm stored in the Broker which is used to provide a cyclic alternation between the resources (VMs). Although very useful and of easy implementation, the systematic pattern of simulations created does not include situations in which other factors should be considered, such as the cases that Quality of Service (QoS) attributes should be applied to client requests. In addition, nothing related to service differentiation, such as cost, energy-efficient use or even workloads with bursts is considered. When using the original CloudSim it is necessary to customize the default characteristics (VMs, hosts, and other components) in order to reach the configuration details, needed to undertake more interesting evaluations and validations on the use of cloud computing resources. In fact, CloudSim was developed for this type of application, i.e., it provides an API with the essential resources, and all the particularities required in certain simulation situations should be added by developers.

## 4. An Extension for the CloudSim Architecture

Several recent studies have used simulation to evaluate the performance of different scenarios in Cloud Computing [10, 13, 15, 17] and most of them based on simulation use CloudSim. A load is imposed on the components of the cloud computing system by sending requests (cloudlets) to be executed. Many works using CloudSim standard API consider that the workload is submitted by DatacenterBroker entity, thus all the previous details of the sending process to the request arrival to the Broker are discarded (such as information about the source client and network latency). This is because the CloudSim standard API has components centered on elements contained in the Cloud Provider.

For many research approaches in cloud computing, the proposal of the CloudSim API is sufficient, however, when it is necessary to incorporate the cloud computing client entity, the simulator does not have this component available. Few published works consider an approach that includes a greater focus on the perspective of the client [15].

There are many scenarios where the importance of the client entity is evidenced. For example, in a scenario where the clients are geographically distributed, located outside of a cloud Data Center, it must be necessary to consider the network latency before the arrival of the request to the broker because this can impact on the service performance. In addition, the client entity can represent various situations that makes the simulation more realistic, such as sending (upload) or receiving (download) a file using the Internet, sporadic HTTP (Hypertext Transfer Protocol) requests to websites, portals or e-commerce, sending individual or group requests of Web services and scenarios where the workload is in the form of bursts. The phenomenon of bursts in workloads is characterized by the unexpected increase in service requests submitted to the system, which results in peaks because of either irregular temporal intensity of arrivals, or intensity of services demands imposed to the system. The performance of the service may be significantly affected by the workload that exhibits high variability in a given space of time [18, 19, 20] and therefore need to be considered in the workload model.

An entity that runs in simulation time and interacts with other entities through the exchange of messages and execution of simulation events must be included in CloudSim so the clients that represent the situations exemplified can be created.

This paper addresses the implementation and evaluation of such a new entity in CloudSim, which is inherited from the SimEntity class, interacts with DatacenterBroker entity (also changed with new attributes and methods), and allows the instantiation of multiple clients with different execution characteristics. The way each client instance executes, i.e., how it sends its requests, was named as the submission of workload and is divided into three categories of execution: vector of requests, requests in real time and group requests. These three categories incorporate in their implementation a waiting time (think time) between submissions of two successive requests, representing the interval between arrivals (inter-arrival time).

The Client class also enables the combination of instances of clients with any CloudSim topology implemented in the network and can interact with a fixed latency setting obtained by methods of NetworkTopology class or as part of a more complex network graph by the use of Brite tool [21]. Another distinguishing aspect of the new client entity is its ability to send requests to different services hosted in the VMs of the cloud providers. This aspect is interesting because it enables the implementation of many different scenarios in which the client can use one, more than one or a composition of services, by means of the Broker differential negotiation processes. These different services can be generated uniformly or randomly by simply invoking methods that perform this configuration on the client entity. The source codes of this new extension implemented for Cloudsim architecture are available on GitHub repository at *https*:*//github*.*com/BEQoSCoders/BEQoS*.

**Fig 1:**
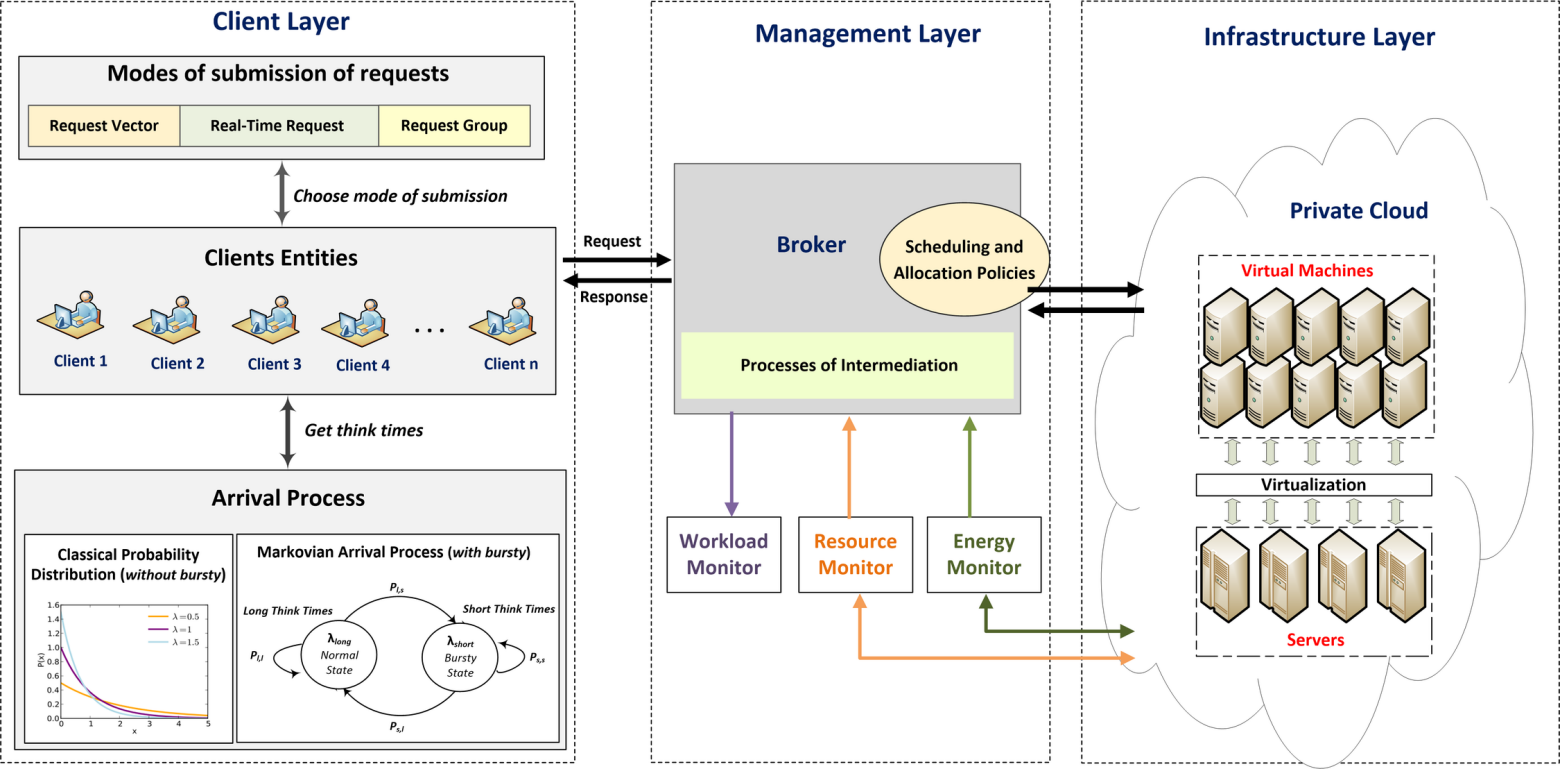
Architecture for the approach considering the new client entity. This figure shows the architecture of the simulation organized in layers, considering the new client entity.

Fig 1 shows the architecture of the simulation organized in layers, considering the new simulation entity. The architecture consists of three layers:

- *Client Layer*: the client entities, modes of submission of requests and workload models that implement the request arrival process are implemented in this layer. Client entities send the concurrent requests to the Broker through one of the request submission modes. The request arrival process is modeled to represent situations with or without bursts. Under no bursts, arrival processes can be generated through classical distribution functions, such as exponential, uniform, Pareto, among others. For situations in which bursts are considered, the workload is modeled by the Markovian Arrival Process (MAP) of two states. All these features are presented in Section 5;
- Management Layer: this layer is responsible for task scheduling, allocation and provisioning of requests in the Data Center and monitoring of workloads and resource consumption. The Broker entity acts as an intermediary agent and has policies for differential treatments for the scheduling of tasks submitted individually or in groups. The service requests submitted by clients are received by the Broker, which forward them to the cloud resources. The scheduling policy, responsible for allocating the requests on the VMs, selects all VMs that have the service asked and then verifies which of the selected VMs are idle or, if there is no idle, verifies which one has the smallest waiting list. Thus, the request is scheduled to an idle or a less overloaded VM that has the requested service. The entities responsible for system monitoring are also located in this layer. The workload monitor collects the rate and the amount of requests sent to the Broker at defined time intervals, set prior the simulation. The resource monitor notes the resource consumption of a data center (percentage of millions of instructions Per Second (MIPS)) and the energy monitor captures information about the power demand (in watts) consumed by the Private Data Center;
- Infrastructure Layer: physical and virtualized cloud computing resources are found in this layer. The cloud computing environment implemented adopts private cloud model, i.e., the entire infrastructure and the cloud services are available for a single organization and may be owned or outsourced.

## 5. A New Client Entity

This section addresses the way new entity considers different types of clients arriving in the cloud computing system being simulated. Three different ways of request submission to the cloud were implemented, as follows:

- *Request Vector Client*: this mode of submission was implemented using an array of cloudlets that is generated after the creation of each client instance. Once instantiated, the client will either send requests successively and without intervals or wait for time intervals called think times (will be explained in Section 5.1). This type of client enables the simulation of several types of applications on the client side, such as transmitting of audio or video streams, uploading a large file, sending e-mails, and so forth.
- *Real-Time Request Client*: in the original CloudSim, the requests that arrive in the Broker are immediately sent to the VMs. It is similar to the client sending all the requests at once for cloud system, and for the rest of the simulation time just waiting for the responses. As known, it does not occur in the real world. The Client implementation proposed provides a type of client component configuration in which requests are sent in a so-called real-time simulation. Instead of sending all requests at once, the client will send them according to the progression of the time of the clock simulation (clock tick) available in CloudSim. This type of client enables the creation of more realistic simulations, as the client behavior becomes more similar to that observed in real environments. Additionally, with the inclusion of the wait time between requests, represented by the think time, an even more reliable behavior toward real users is added to the client.
- *Request Group Client*: most simulations reported in recent studies use the method of sending individual requests to the Broker, hence to the VMs of the data centers. Although this approach represents a large part of the researchers needs, alternative situations such as the use of groups of requests must be implemented. It was developed a way of operation for clients in which groups of requests are sent each time a message has been submitted to the Broker. Therefore, it is possible to set the desired number of requests and ask the client to create groups and send them during the simulation time. Requests in groups may or may not be dependent on the execution order of the tasks of the services requested. By using abstraction, this implementation enables the simulation of different types of client applications using groups of requests, i.e., requesting Web services by composition, and requests for execution of parallel tasks, among many other possibilities. In each group of requests, the client can be configured to request different services in each cloudlet, so that the simulation performs as if the customer was asking for "collections" of services.

### 5.1. Request Arrival Process

The novel client entity enables to generate a waiting interval between requests, called a think time, which refers to the time interval between the submissions of two successive requests from the same client [22,23]. This implementation enables the representation of cloud computing scenarios where to occur bursts of requests. The phenomenon of bursts in workloads is characterized by the unexpected increase in service requests submitted to the system. This leads to irregular temporal peaks in the intensity of the requests arrivals from clients or the intensity of services demands imposed on system resources. These workloads typically arrive in the system with high variability and may cause performance bottlenecks in service times that are difficult to predict. Therefore, the phenomenon of bursts [19, 20, 24] must be considered in the workload model adopted to evaluate the performance of services running on the cloud environment.

Two models can generate samples of think times. In the first, samples of think times are generated following an exponential distribution representing a scenario where there are no bursts in the arrival of requests process. In the second model, the generation of the think times is modeled to represent situations with bursts [20, 25]. In this case, the bursts are modeled by a dispersion index [26] that models the intensity of the burst and uses a MAP with two states to regulate the arrival rate of requests. MAP can be seen as a mathematical model of time series and provides variability at different levels as well as effects of temporal locality.

**Fig 2:**
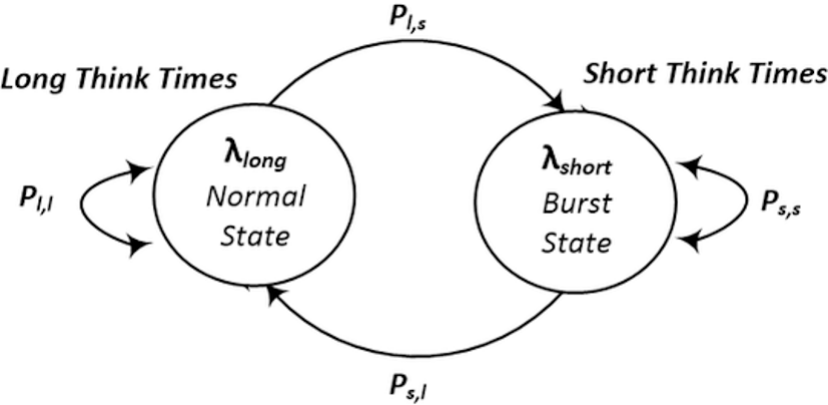
Transition MAP Diagram–Adapted from [20]. Example of a two-state MAP process (Normal State and Burst State) to control the requests arrival rate in the system.

Fig 2 illustrates an example of a two-state MAP process to control the requests arrival rate in the system. The state without bursts (Normal State) is responsible for generating a sequence of long think times, associated with normal traffic periods. The state with bursts (Burst State) is responsible for generating a sequence of short think times, generating smaller intervals between successive requests, thus resulting in bursts in the process of the requests arrival.

The long and short think times are generated with the average rates of λ_*long*_ and λ_*short*_ respectively. In order to ensure a correlation between different events, after the generation of a new sample of think times, the model has a probability *p*_*s*,*s*_ that two consecutive think times are short and *p*_*l*,*l*_ that they are long. The probabilities *p*_*l*,*s*_ = 1—*p*_*l*,*l*_ and *p*_*s*,*l*_ = 1—*p*_*s*,*s*_, determine the changes between the states. Thus, the model is able to represent the duration of traffic peaks and the bursts oscillations within a single peak.

#### 5.1.1. Mathematical Description of a MAP

A MAP process of order *n* (where *n* is the number of states) is defined by two *n*x*n* square matrices, represented by D_0_ and D_1_. The matrix D_0_ + D_1_ is the generator of a Continuous-time Markov Chains (CTMC). Consider that the behavior of transition of the states of a MAP of two states is defined by two matrices {D_0_, D_1_}, where [19] [27]:

$${\text{D}}_{0}=\left[\begin{array}{cc} -\lambda_{i} & 0\\ 0 & -\lambda_{j} \end{array}\right]\;{\text{D}}_{1}=\left[\begin{array}{cc} \lambda_{i}p_{ii} & \lambda_{i}p_{ij}\\ \lambda_{j}p_{ji} & \lambda_{j}p_{jj} \end{array}\right]$$ (1)

MAP process can be characterized by the parameters {λ, *P*}, wherein λ = (λ_*i*_, λ_j_), and by the matrix of probabilities ***P*** {*p*_*ii*_, *p*_*ij*_*; p*_*ji*_, *p*_*jj*_}, where the infinitesimal generator matrix *Q* of the CTMC, can be obtained by the sum of D_0_ and D_1_:

$$Q=D_{0}+D_{1}=\left[\begin{array}{cc} -q_{i} & q_{ij}\\ q_{ji} & -q_{j} \end{array}\right]$$ (2)

And the steady state probability vector of the Markov process can be obtained using Eq 3, which follows the mathematical approach presented in [28] for the two states Markov process:

$$\pi ={[\pi }_{1},\pi_{2}],\;where:$$

$$\pi_{1}=\frac{1}{q_{i}+q_{j}}\;q_{j}\;,\;\pi_{2}=\frac{1}{q_{i}+q_{j}}\;q_{i}$$ (3)

$$\pi Q_{MAP}=0\;|\pi |=1$$

Being **|π|** the sum of the values of the steady state probability vector ***π***. In this way, the value of ***π***_***i***_ represents the steady state probability of the arrival process being in state ***i*** and the ***π***_***j***_ represents the steady state probability of the process being in state ***j***.

#### 5.1.2. Fitting of MAP parameters

The main measures used to characterize the bursts modeled by a MAP, which are considered to define the parameters of MAPs models in this work, are described below:

- **Average**: is the average (*λ*^*-1*^) of the arrival intervals generated by MAP. MAP average can be obtained by calculating the moments of arrival intervals generated by MAP. For a MAP, the moments can be calculated by Eq 4, considering ***e*** a column vector with size *m* and all positions filled with value 1 (where *m* is the number of states in MAP). The average is the first moment, i.e., *k = 1*:

$$E\left[X^{k}\right]=k!\left(\pi \;D_{1}\right){\left(-D_{0}^{-1}\right)}^{-K}\left(\frac{e}{\pi \;D_{1}e}\right)$$ (4)

- **Squared Coefficient of Variation (SCV):** is the variation coefficient square of the arrival intervals. This measure quantifies the variability of a sequence of random values. Distributions with SCV < = 1 are considered with low variability. For an exponential distribution, for example, the SCV value is equal to 1, which indicates a low variability. As the SCV value increases, the more variable the sequence of random values becomes. Whereas σ^2^ is the variance and *σ*^2^ = *E*[*X*^2^] − (*λ*^−1^)^2^, the SCV can be obtained by Eq 5:

$$SCV=\frac{\sigma^{2}}{{\left(\lambda^{-1}\right)}^{2}}$$ (5)

- **Dispersion Index (I):** represents the bursts intensity level, which can arrive in shorter or longer periods through time. The higher value of I means that the intensity of the burst is more severe and therefore can significantly affect the service performance. When no burst occurs the value of I is equal to SCV, and for an exponential distribution, I = SCV = 1. Considering yet that ***λ*** represents the average arrival rate of MAP and *λ = π*.*D*_*1*_.*e*, the index of dispersion can be obtained by Eq 6:

$$I=1+2(\lambda -\left(\frac{\pi D_{1}}{\lambda }\right)\left(Qe\pi {)}^{-1}\left(D_{1}e\right)\right)$$ (6)

In this work, the MAP of two states is parametrized by the average of MAP (*λ*^*-1*^), by the index of dispersion (*I*) and by the square of the coefficient of variation (SCV) in order to create sequences of peaks with different intensities and durations. It was used a mathematical description of a MAP process and the support of tools such as MATLAB (*MATrix LABoratory*) software [29] and the KPC-toolbox [30], which is a collection of MATLAB scripts focused on parameterization and analysis of MAP.

**Fig 3:**
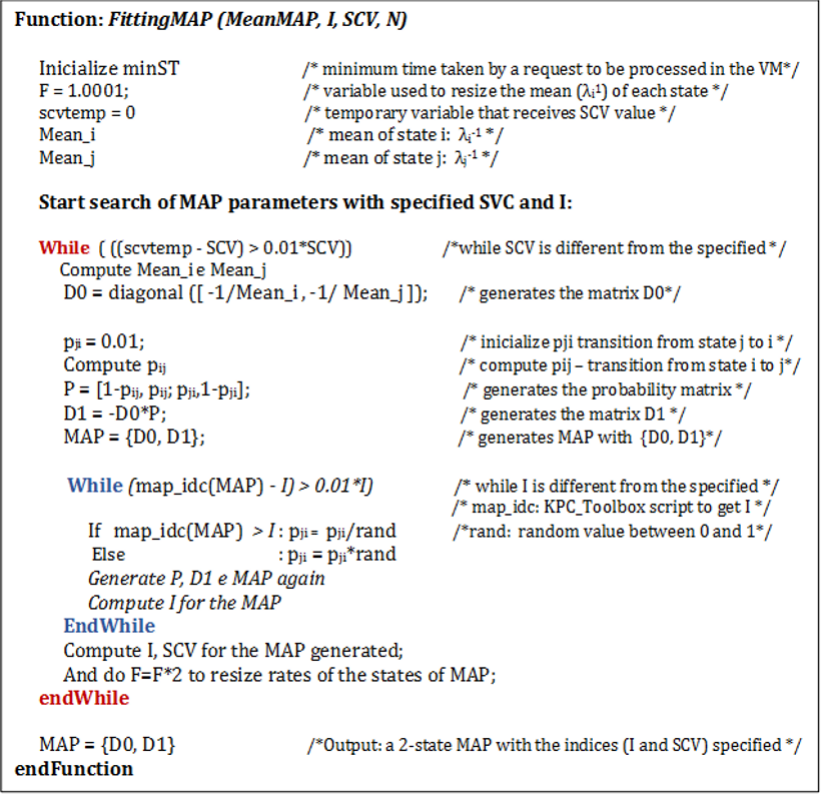
Pseudo Code–defining the parameters of the MAP. Script for fitting the parameters of a MAP to model bursts in the arrival process, considering the main measures as average, I and SCV of the MAP.

A script was implemented to determine the MAP parameters (*λ*_*i*_, *λ*_*j*_, *p*_*ii*_, *p*_*jj*_) to model bursts in the arrival process. These parameters are obtained from the input arguments: MeanMAP (*λ*^*-1*^), I, SCV, and N (number of concurrent clients submitting requests to the system). The proposed approach to define MAPs parameters considered in this study is described in the script presented in Fig 3.

This pseudocode considers that the input arguments (MeanMAP, I, SCV, N) are provided by the user and that the average of arrival intervals are initially defined by Eqs 7 and 8 (adapted from [20]), where *MinST* is the minimum time for a service to be completed in a VM.:

$$\lambda_{j}^{-1}=\frac{MinST}{f}$$ (7)

$$\lambda_{i}^{-1}=\text{max}\left(f\lambda^{-1},fNMinST\right)$$ (8)

This time was obtained based on previous experiments, considering measurements of the minimum time for the services execution in VMs of the data centers described in Section 6. The variable *f* > = 1 is a free parameter used in the resizing of the average time in the states *λ*_*i*_^*-1*^ and *λ*_*j*_^*-1*^. Thus, assuming that the initial value of *f* = 1,001, the average in state *i* (*λ*_*i*_^*-1*^) initially receives a higher value to complete requests in VMs (to generate long think times), while the average in state *j* (*λ*_*j*_^*-1*^) is initially set to a value close to the minimum time for a service be completed (to generate short think times). It is important to remember that the averages of the two states are resized in each search for a parametrization that meets the values previously specified (MeanMAP, I and SCV).

Just as the average of the states and based on the work presented in [20], the transition probabilities from state *i* to *j* can be obtained by Eq 9:

$$p_{i,j}=p_{j,i}\;*\;\frac{Mean_{-i}-MeanMAP}{MeanMAP-Mean_{-j}}$$ (9)

where *Mean*_*_i*_ and *Mean*_*_j*_ are the averages of the times generated in the states *i* and *j*, respectively. The probability of transition from state *j* to *i*, *p*_*j*,*i*_ is set to an initial probability of 0.01. However, in the process of setting the MAP parameters, the value *p*_*j*,*i*_ is resized (so that: 0 < *p*_*j*,*i*_ <1). In each iteration, the dispersion index values (I) and the Squared Coefficient of Variation (SCV) are checked. The search is completed when it is found a MAP with I and SCV that are within 1% of the indices specified.

### 5.2. Client Events

The implementation of new entities enables new components to be introduced into the simulation scenario and also the interaction with the other entities of cloud computing through event-oriented communications. In the implementation of the client entity proposed in this paper, several events representing interactions between the entity and the Broker or CloudSim core management were inserted:

NEW_CLIENT_ARRIVAL: invoked by the entity simulator at the boot time. When client is started, this event sends an information message to the output terminal, initializes a client entity that will start sending cloudlets and starts (if enabled) the generation of think times;

CLIENT_SENDING_CLOUDLET: refers to all events in which the client sends its requests, whether individual or group. This event is where it is verified how the client was configured and then the tasks to be sent to the Broker are generated;

CLIENT_RECEIVING_RESPONSE: occurs every time the client receives a. Upon receiving notification of the response of a cloudlet the client turns to the state of sleeping (the method *pause* is used for this) during a finite time delay of a think time;

CLIENT_FINISHED: finalizes the activities of the client entity. It is invoked by the simulator when the entities are closed, i.e., when it is called the *shutdownEntity* method. This causes the entity to stop the calls to the run method and print a message notifying the completion of the client.

Although these events are the basis for the operation of any client, nothing prevents users from adding other behaviors to that entity by adding new events.

### 5.3. Methods of Sending Requests

The original CloudSim method of sending requests submits a set (or list) of requests (cloudlets) to the Broker which stores it in an internal list called Broker *cloudletList*. At the beginning of the simulation, an internal method is invoked from the Broker and submits the list of cloudlets to the VMs, according to the sequence they were placed in the list. The mode of transmission of requests uses a cyclic rotation algorithm, alternating the VMs to balance the load imposed on the data centers available.

The client entity has some particularities that allow other behaviors in simulation time. The characteristics are associated with the method (or mode) for forwarding the requests so that they can be sent "in real-time simulations", i.e., they are sent during the progression of the internal simulator clock (known as clock tick) as if they were being sent in small portions by clients. This configuration is called *REAL_TIME_REQUESTS*. When sending a request, the client entity waits for a random time (or not) in sleeping mode and then returns to operation performing a wake-up and creating a new task to be sent. This acts as an intermittent cycle. In this mode of transmission, the simulation should stop by simulation time and the Broker waits indefinitely for the arrival of new requests.

The sending method of the new client implementation also has an interesting feature related to the ability to create simulation scenarios that contemplate the arrival of requests in bursts, causing various realistic behaviors of cloud systems that are tested under stress due to large workloads. Two methods are proposed for the implementation of the client think time behavior. The first one is the *getNextThinkTime*, which uses a probability distribution defined prior to the simulation (as exponential, uniform, Pareto, among others) to generate think time samples. The second method is the *getNextThinkTimeMap* used to represent situations of bursts in the process of arrival of requests. The sequences of think times are generated from the MAP class of two states, as shown in *Section 5*.*2*. The presence of bursts in the arrival process shows some specific properties of this type of workload [20]. One of the features is clients act in aggregate form, thus the process of requests arrival and think times cannot be randomly generated and independent of each other to capture the essential characteristics of this type of workload. Therefore, samples of think times are generated by a MAP class of two states that are shared among all clients. This immediately solves the question of independence between requests of different clients.

## 6. Experimental Design

Experiments were conducted to demonstrate that the adoption of the new client entity proposed enables the creation of scenarios in which clients influence the simulation and make the simulated environment more realistic. Three sets of experiments were performed to achieve this goal, as shown in Table 1.

**Table 1:** Sets of Experiments.

Sets of Experiments	Factors	Configuration Levels
**1**	Models of Submission	■ Request Vector
		■ Real Time Request
		■ Group Request Dependent
		■ Group Request Independent
		■ Standard CloudSim API
**2**	Request Arrival Process	■ Without Burst
		■ With Burst
**3**	Network latency between	■ Homogeneous latency
	Client and Broker	■ Heterogeneous latency

The first set refers to the mode of request submission used. Three models were considered: single submission of requests (with two possibilities: request vector and real-time requests), submission of requests in group (requests with and without dependence on each other) and model of requests that follows the pattern of the CloudSim API, totalizing five options. For these experiments, the simulation environment has been configured to run with a network latency of 500 milliseconds between Client and Broker. This set of experiments aims not to compare the results from different modes of submission but show that it is important to consider the way that the requests arrive to be executed since it may have a large influence on the response variables.

The second set of experiment is associated with the request arrival process, i.e., the generation policy of think times to be adopted in situations with or without bursts. For the execution of these experiments, the arrival without bursts was configured with think times following an exponential distribution with mean think time *E[Z]* of 7 seconds. The workloads with bursts sequences of think times were generated using the MAP class of two states [20, 25], where the parameters of MAP were defined by mean (*λ*^*-1*^) of 7 seconds, index of dispersion (I) equal to 4000 and SCV equal to 7, as described in Section 5.1.2. Two other options can be evaluated. In the first one, the time to send the next request (think time) starts counting immediately after sending the previous request, and in the second option, it starts counting only when receiving the complete response of the previous request. For these experiments, the simulation environment has been configured to run with individual modes of submission, using request vector and a network latency of 500 milliseconds between Client and Broker. This study aims to evaluate how the bursts in the arrival process of the requests cause a significant impact on the performance of services running in a private cloud.

The third set of experiments considers the network latency between the Client and the Broker. Two options for the network latency were defined: homogeneous and heterogeneous. The submission mode considered in these experiments is the request vector. The homogeneous latency was configured with a fixed amount of 500 milliseconds and the heterogeneous latency with random values generated in the range of 0 (zero) to 2000 milliseconds. This study allows evaluating the system behavior when considering clients in similar or different geographic locations.

All experiments involved 100 concurrent clients responsible for the submission of requests for different services over a simulation time of 5000 seconds. Each client sends a fixed amount of requests over the time of simulation (100 requests) in the case of experiments that adopt the submission modes: request vector and group request. In the case of submission mode in real time where the sending process occurs throughout the simulation time, a variable amount of requests is considered. Each request has a size or demand of 100,000 Million Instructions (MI) service for individual modes of submission. For the mode of submission group, it was considered groups of 4 requests, each with a service size of 25,000 Million Instructions.

Additionally, the data center considered is composed of 10 servers (hosts) physically homogeneous. The physical configuration of each server unit exhibits six processing units or cores x86 architecture, with a capacity to process 10,000 Million Instructions Per Second (MIPS), 24 gigabytes (GB) of main memory, 147 GB of storage units and 16 network cards of 10 gigabit/second transmission rate each. Similarly to physical servers, virtual machines were considered homogeneous and managed by the Xen hypervisor [31]. VMs were instantiated in all 40 experiments, each with one processing unit x86 architecture with a 10,000 MIPS processing capacity, consuming 4 megabytes (MB) of primary memory. In addition, 5 Web services were randomly distributed among VMs, where each VM was responsible for the execution of two different Web services.

Some aspects related to the performance of the service, including the following response variables, are considered:

- *Average response time*: time interval between sending the request by the client and completion of the response processed by the cloud. It´s the sum of the average system time and the network Latency.
- *Average system time of requests*: the sum of the time spent on the VM queue and the time of processing the request on the VM.
- *Average throughput*: the average rate at which requests are served by private cloud, measured in requests per second.

During the simulation, the number of arrivals of requests monitored at time intervals of 7 seconds, and the resource consumption percentage of the private cloud monitored at time intervals of 1 second, were analyzed. Samples of such monitoring are provided in the next section.

## 7. Analysis of Results

In this section, the results obtained from the execution of the experiments are presented. For all experiments, 10 runs were used to determine the average, the standard deviation and the confidence interval of 95% according to *T-student* Table.

### 7.1. Modes of Submission

**Fig 4:**
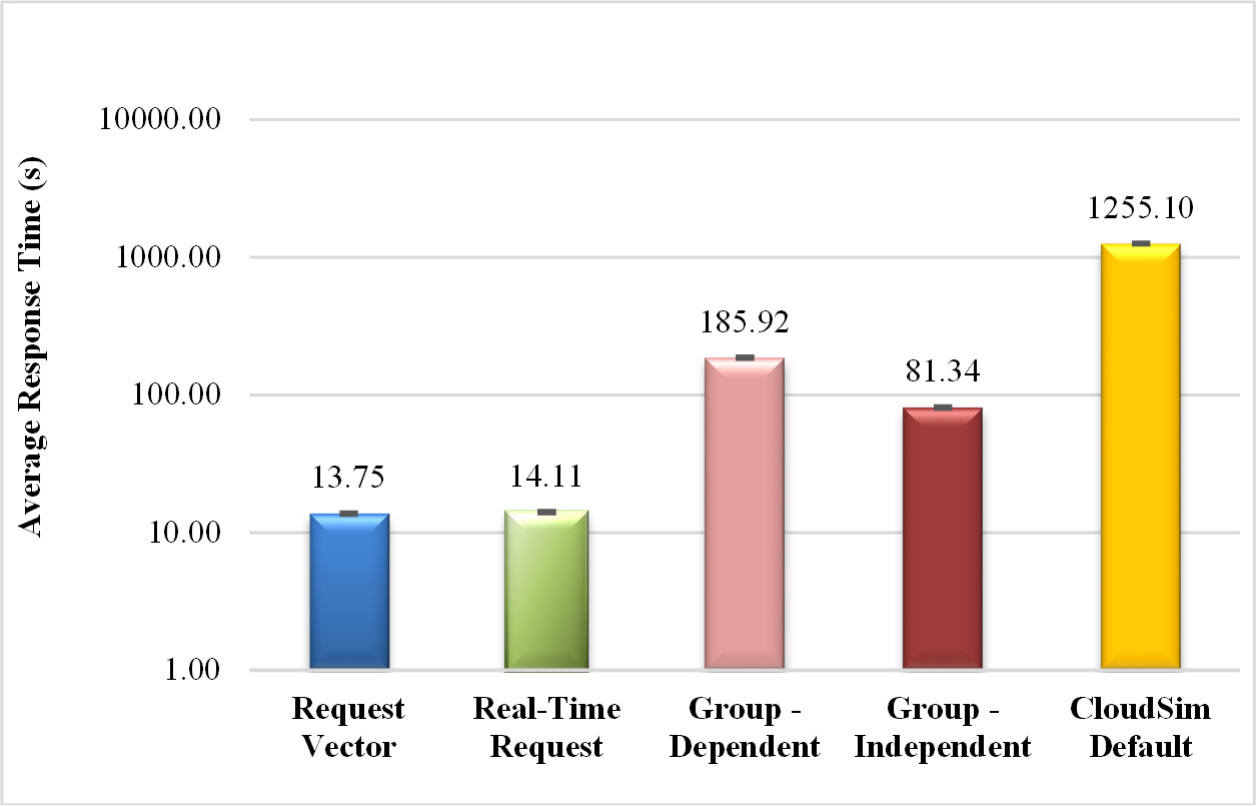
Average response time in seconds for different ways of submission. This graph shows the way that the requests arrive to be executed in the cloud influences in the average response time.

The results of the experiments that consider different modes of submission of the new client entity as well as the standard submission of CloudSim are presented in this section. Fig 4 shows the average response times for each experiment.

As can be seen, the submission modes have different average response times because each of them has different characteristics. The results obtained for the individual modes of submission (request vector and real-time request) show very similar results. This behavior is due to the fact that, although they have different implementations, the two individual submission modes are equivalent. That is, in terms of implementation, the first one considers the request vector and the second one the creation of the requests in real-time. In terms of execution, each request submission is performed in the same way, one at a time throughout the simulation.

Modes of submission in group (dependent and independent requests) show greater average response times in relation to individual modes of submission because the requests are sent in groups, thus, the VMs queues become larger. The submission mode of groups with dependent requests has an average response time greater than groups with independent requests. This occurs because the dependent requests have an execution order and they need to wait for the completion of its predecessor, which does not happen in a group with independent requests since there is no order and it may execute in parallel.

Fig 4 also highlights the average response time using the default submission in the original CloudSim. Note the significant increase in this response variable. This increase in the average response time is due to the default behavior of CloudSim, which sends all requests at once, at the beginning of the simulation, resulting in an initial burst of demand.

**Fig 5:**
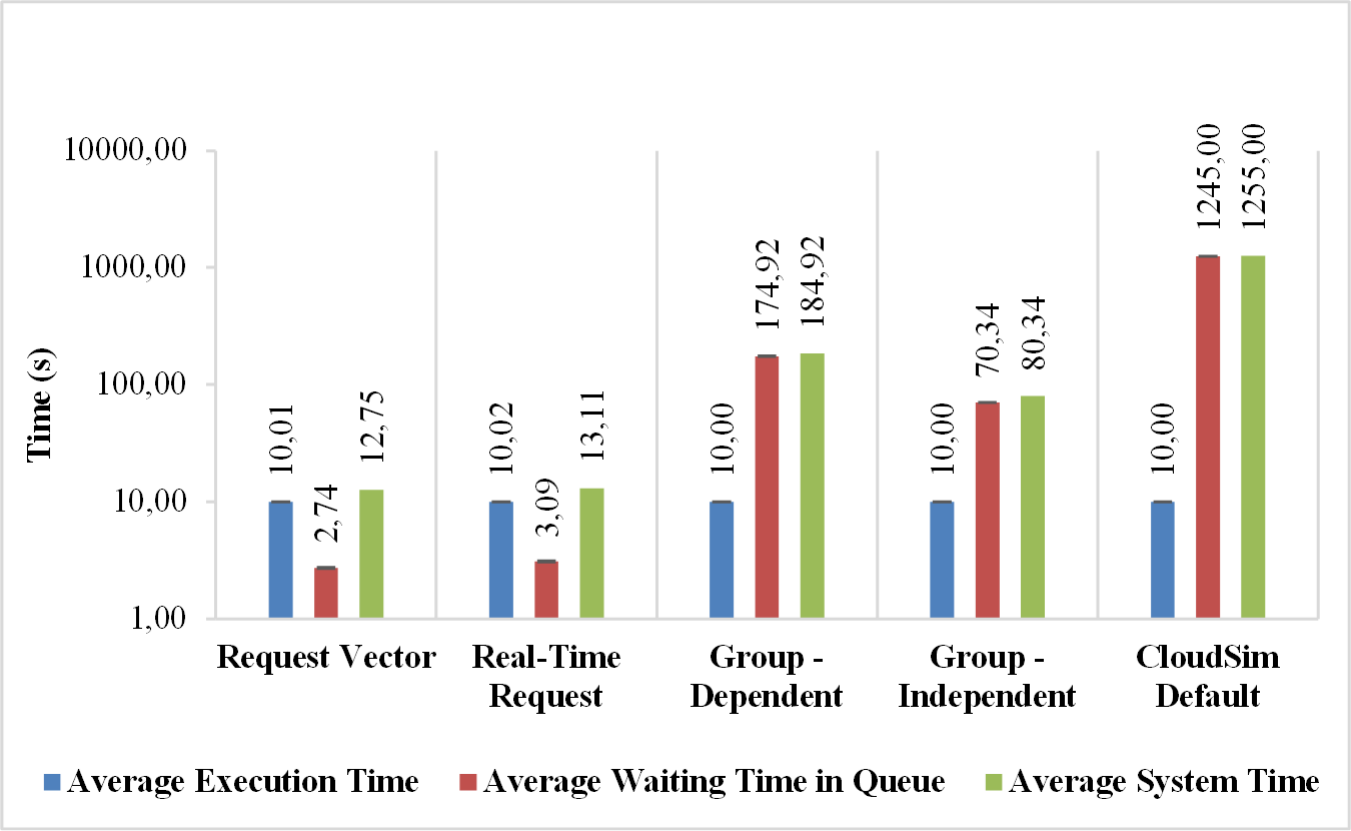
Average system time in seconds for different ways of submission. **E**ach set of three bars considers a way of request submission, for both individual requests or in groups. Each way of submission has three bars, representing parts of the response time.

The results for the average system time are shown in Fig 5. The average system time is split into two components: average waiting time in queue and average execution time, also shown in Fig 5. For all modes of submission, and the standard CloudSim, the average execution times are almost the same. This is because all the experiments considered that the request has a homogeneous demand of 100,000 MI. On the other hand, the queue times in the virtual machines are smaller for the individual requests (around 30% of the execution time) and larger for the other cases.

**Fig 6:**
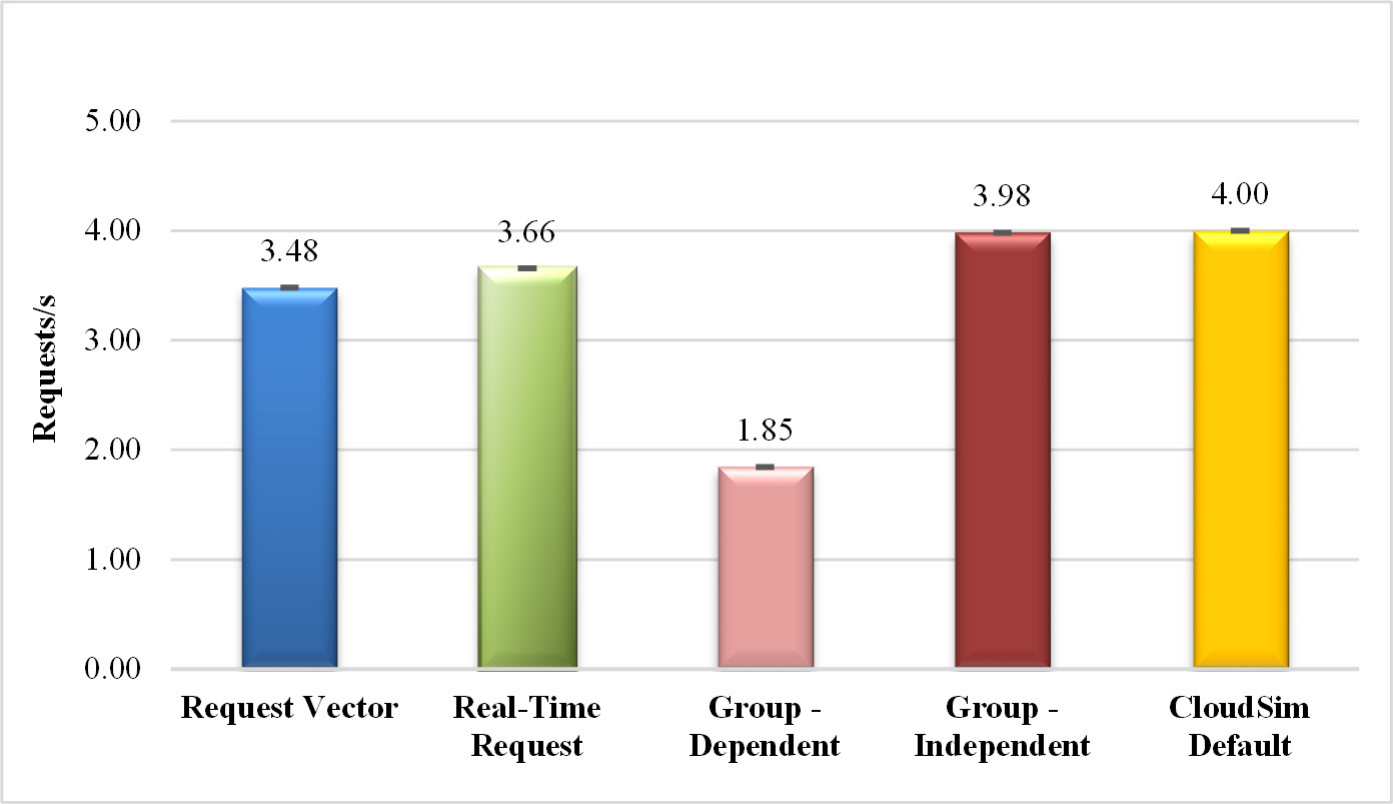
Average Throughput in request/second for different ways of submission. The experiment that consider dependent request group mode presents lowest average throughput, mainly influenced by the synchronization between requests of the group.

Fig 6 shows the results of the average throughput. The lowest average throughput refers to experiments with clients of a dependent request group (1.85 requests / second). The low throughput obtained in the private cloud system is justified by the fact that dependent groups cause a delay in responses due the synchronization between requests of the group. The standard CloudSim model shows a small increase in the average throughput, which is the highest throughput obtained in all experiments. This result was obtained because the CloudSim default submission mode has neither any network latency between client and Broker nor the waiting time (think time) between sending successive requests. This model, however, is not a realistic scenario.

**Fig 7:**
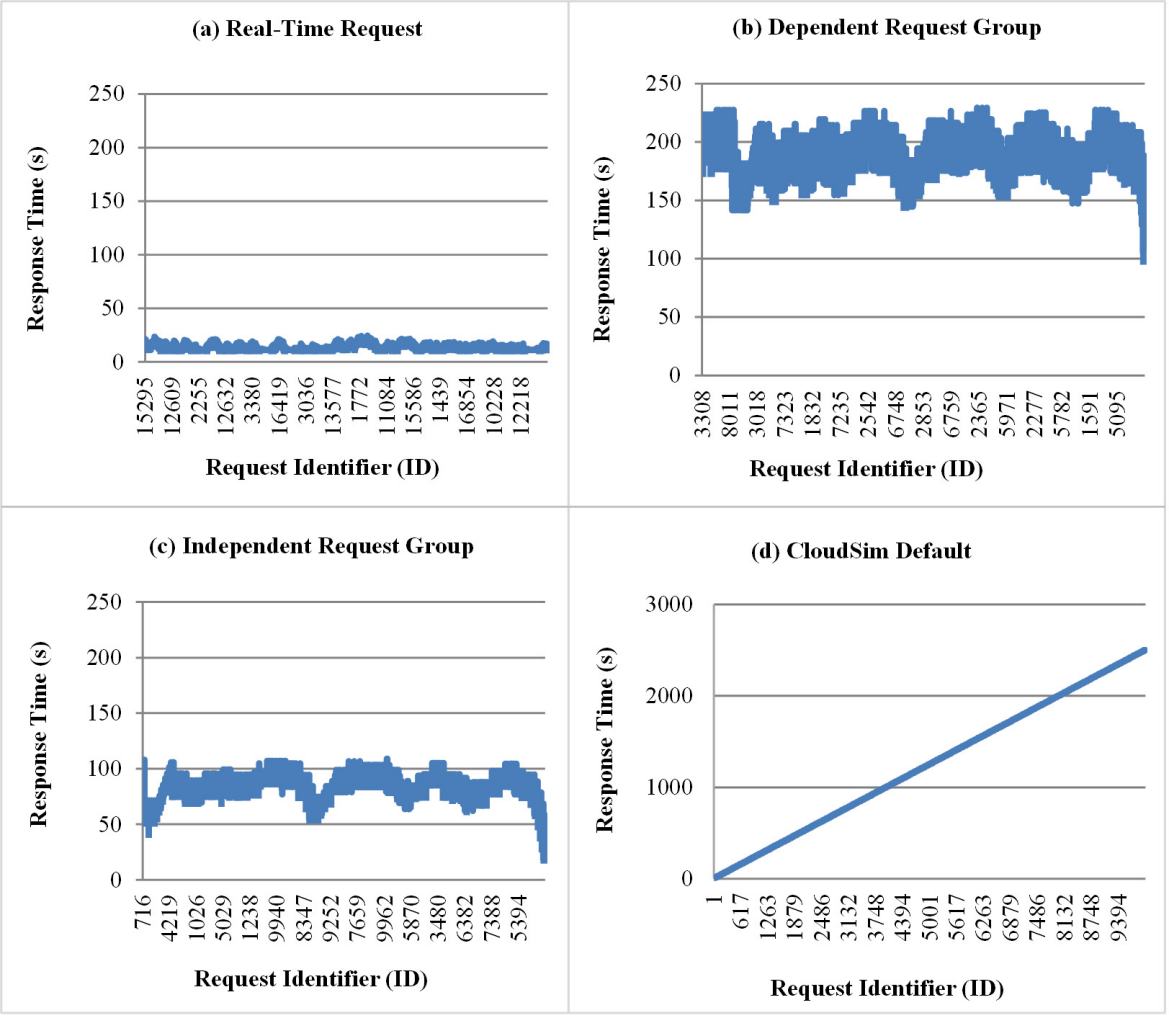
Samples of response times for the different modes obtained of submission. The graphics show the different behavior between client send modes considering the response time and the requests ID during the simulation time.

The following graphs show samples of the execution of one run of experiments considering the individual submission mode of requests (Real-Time Request), the group submission mode (Dependent and Independent Group Request) and the original CloudSim mode. Fig 7 shows the response times for each request submitted to the private cloud. The results reflect the behavior explained above for the average response time, where the modes of submission in group have response times greater than those obtained for the individual modes of submission. In addition, in Fig 7(D), it is observed the response time for the standard model of the original CloudSim that presents a linear growth, since all requests are sent at the beginning of the simulation.

**Fig 8:**
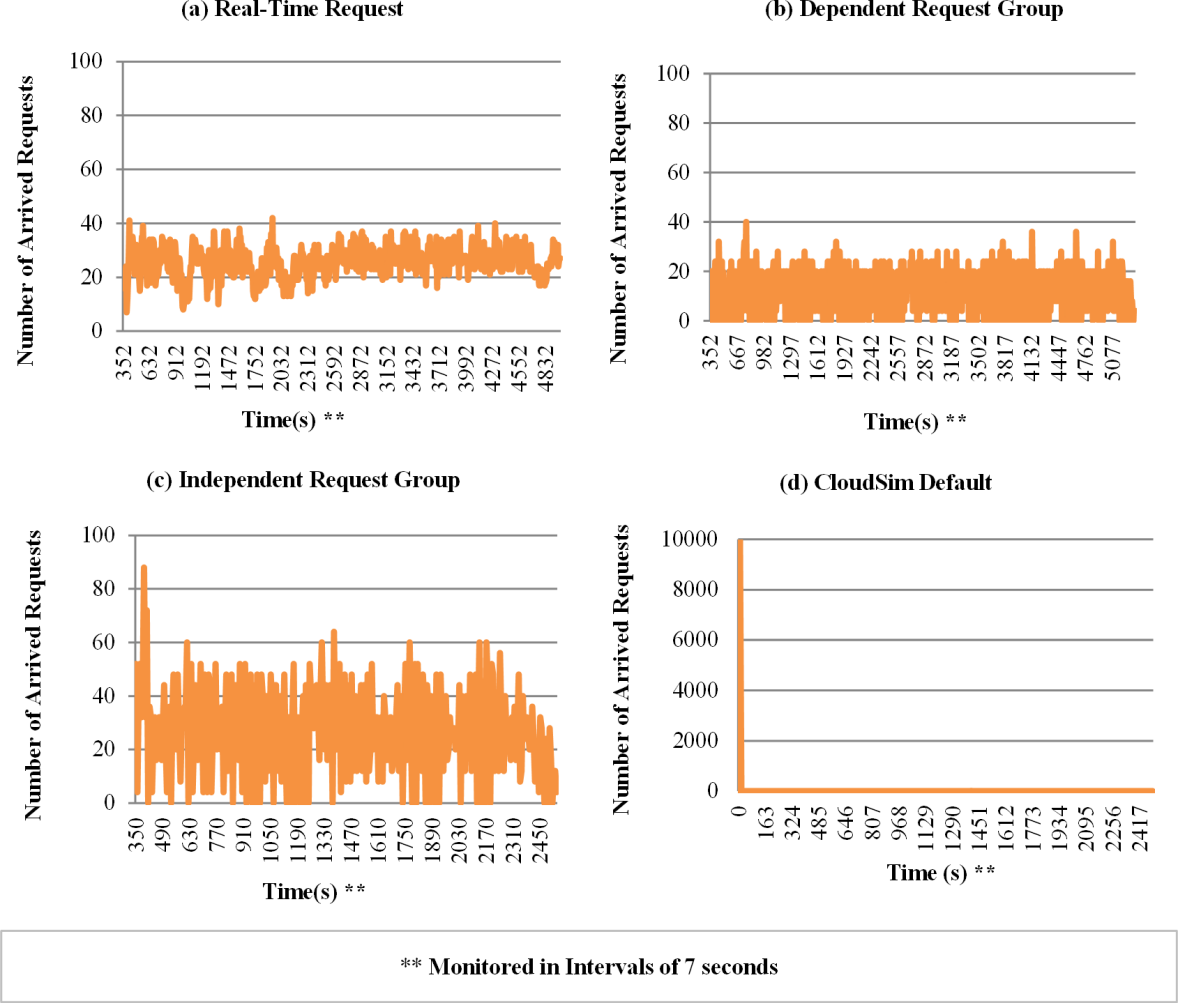
Samples of the number of arrivals of requests for the different modes of submission. The graphic**s** show the number of requests arriving monitored at time intervals of 7 seconds in the simulation.

Fig 8 shows the amount of requests arriving during the simulation time, monitored at time intervals of 7 seconds. It is possible to highlight, as in Fig 8(C), that the results obtained with groups of dependent requests present an amount of the arrival of requests smaller than the observed in the independent group. This is because the effect caused by ordering the execution of tasks (cloudlets). The number of arrivals of requests in experiments with Cloudsim standard mode (Fig 8(D)) shows an initial peak of 10 000 requests in the first moment of the simulation and zero for all other instants of the simulation. This is due to the fact that the CloudSim sends all the workload at once at the beginning of the simulation, as explained before.

**Fig 9:**
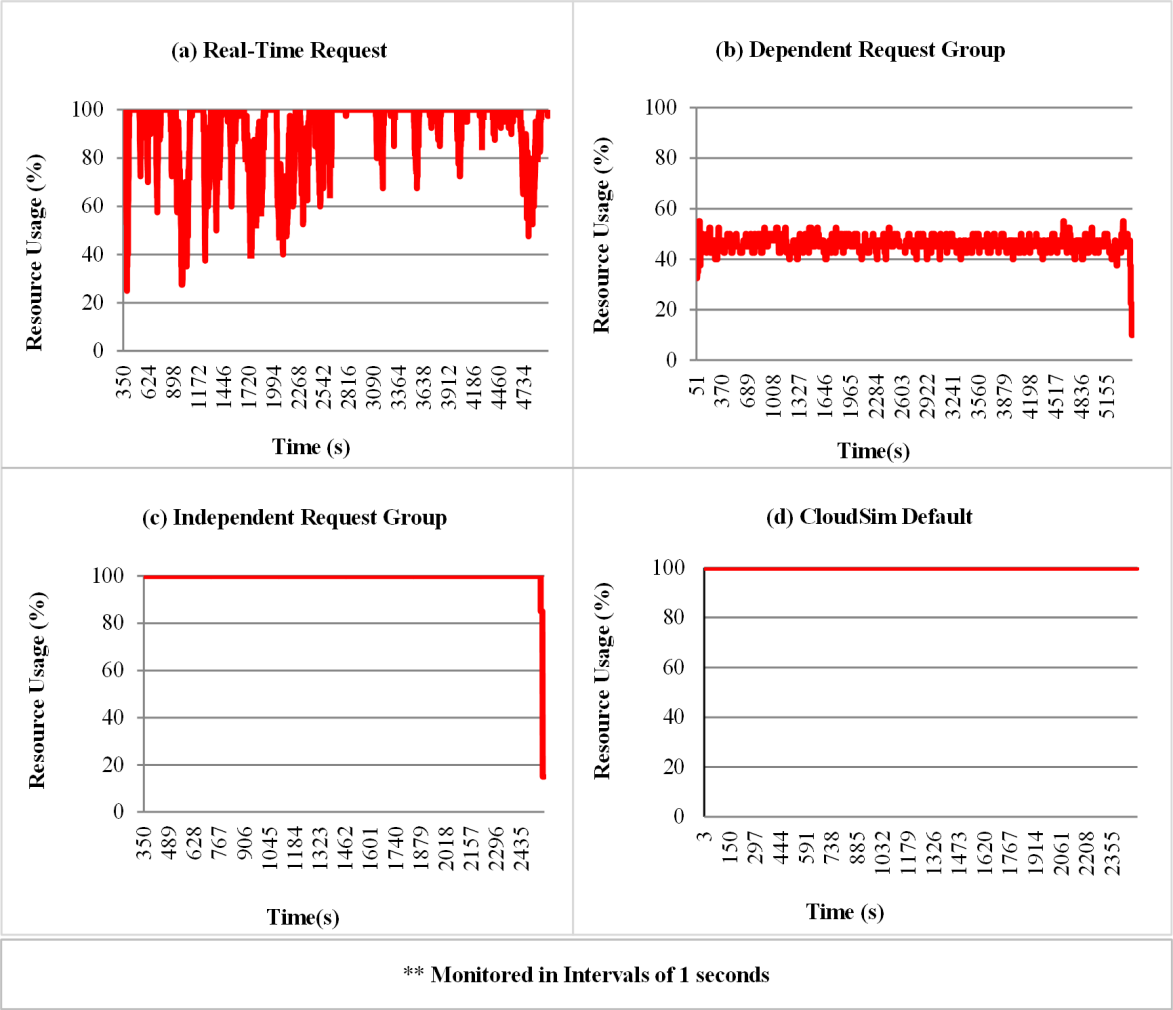
Percentage of resource usage for the different modes of submission. The graphic**s** show samples of the percentage of resource usage, monitored at time intervals of 1 second in the simulation.

The samples for the percentage of private cloud system resources used are shown in Fig 9. The data were monitored at intervals of 1 second. Fig 9(A) shows a high resource consumption in the individual real-time requests and lower resource consumption in groups of dependent requests because of the synchronization of tasks which makes the VMs idle in some periods. The high resource consumption caused by groups of independent requests (Fig 9(C)) is due to the parallel execution of requests. The high consumption of resources evidenced in the standard CloudSim mode of submission (Fig 9(D)) is caused by the initial burst with a large amount of requests, which leaves all resources occupied during almost the entire simulation.

### 7.2. Burst Phenomenon

Systems submitted to workloads in bursts can severely affect the performance of a service provider and lead to a significant overload of the servers, uncontrolled increase in response times and, in the worst case, unavailability of the service. A set of experiments proposed in this study address situations in which two types of workload are considered (workload without bursts and with bursts), considering two configurations of think time (occurring after sending a previous request and initiating after the arrival of the full response of the previous request). The results obtained from the execution of these experiments are presented and explained in the next sections.

#### 7.2.1. Bursty and Non-Bursty Workload with Inter-Arrival Time after the Arrival of the Response of the Previous Request

**Fig 10:**
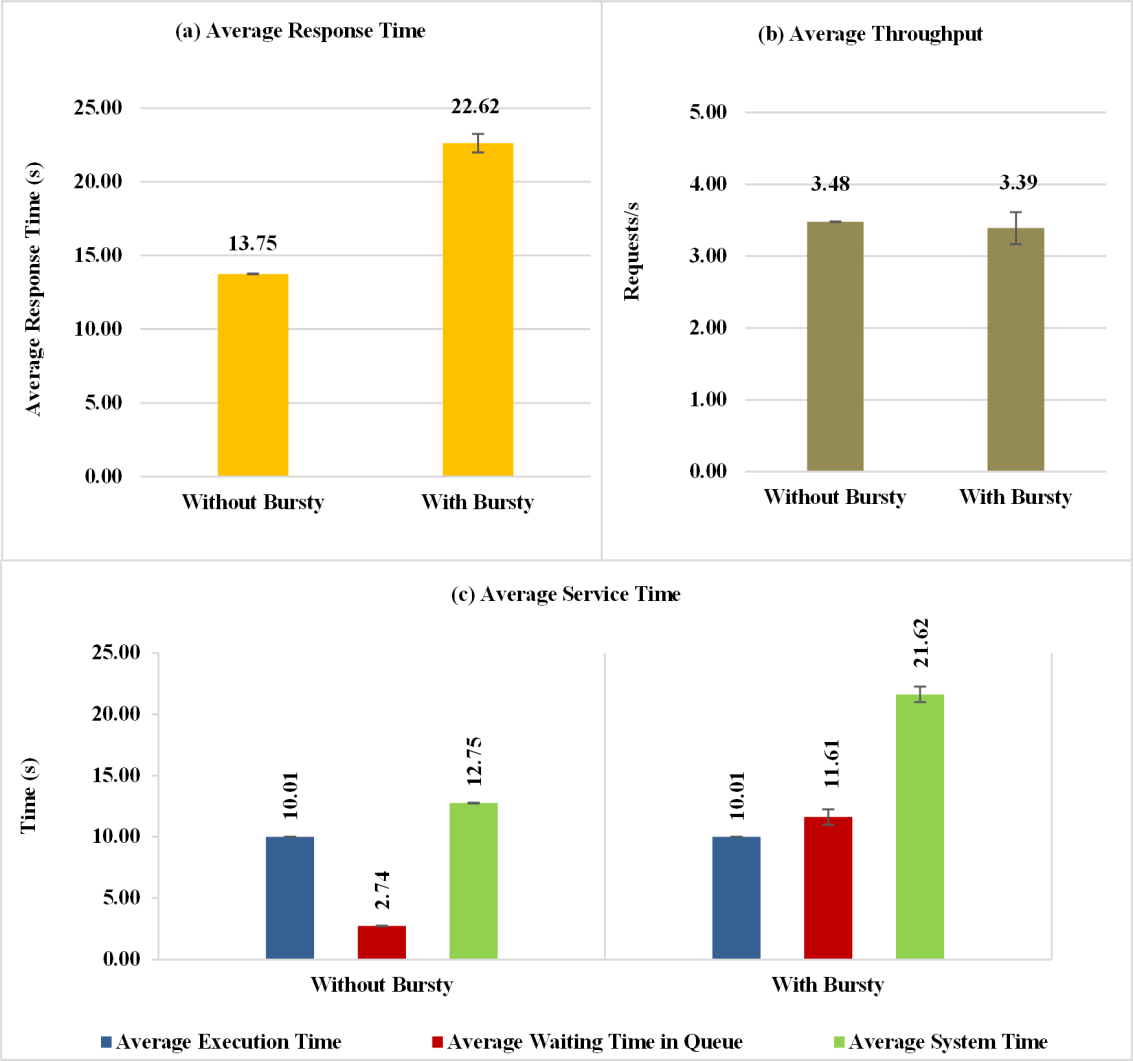
Response Variables considering Bursty and Non-Bursty Workload with think time after response. Experiments that consider workloads with and without burst and using think times beginning after receiving the response from the previous request (a) Average response time (b) Throughput (c) Average service time.

The average response time obtained in the experiments is shown in Fig 10(A). When the workload with bursts is generated, the average response time increases approximately 64% in comparison to situations without bursts. This is because, in certain periods of time, there is an unexpected increase in requests (burst) for service submitted to the resources of the cloud, presenting an impact on the time required to attend these requests.

Fig 10(B) shows almost the same average throughput obtained for the two workloads. The explanation for the values of average response time and throughput should consider the characteristics of workloads with and without burst. In the case without bursts, the requests arrive in a distributed way over the time and a high utilization for the Data Center was obtained (approximately 90%). In the case with bursts, there are periods with many requests arriving that cause a large increase in the queue and in the average response time of the requests and there are periods with few requests that enables the execution of a large number of requests that were waiting in the queue. A large number of requests executed during this period cause an increase in the number of completed requests for workloads with burst and it becomes larger than the case with no burst. This feature makes the throughputs of workloads with and without bursts equivalents.

This impact of bursts in service performance is even more evident in the results for the mean system times, shown in Fig 10(C). When the workload behaves in bursts, the average waiting time in the queue of the VMs and the average system time increase. These results show the presence of bursts in the process of arrival of requests has a negative impact on service performance.

**Fig 11:**
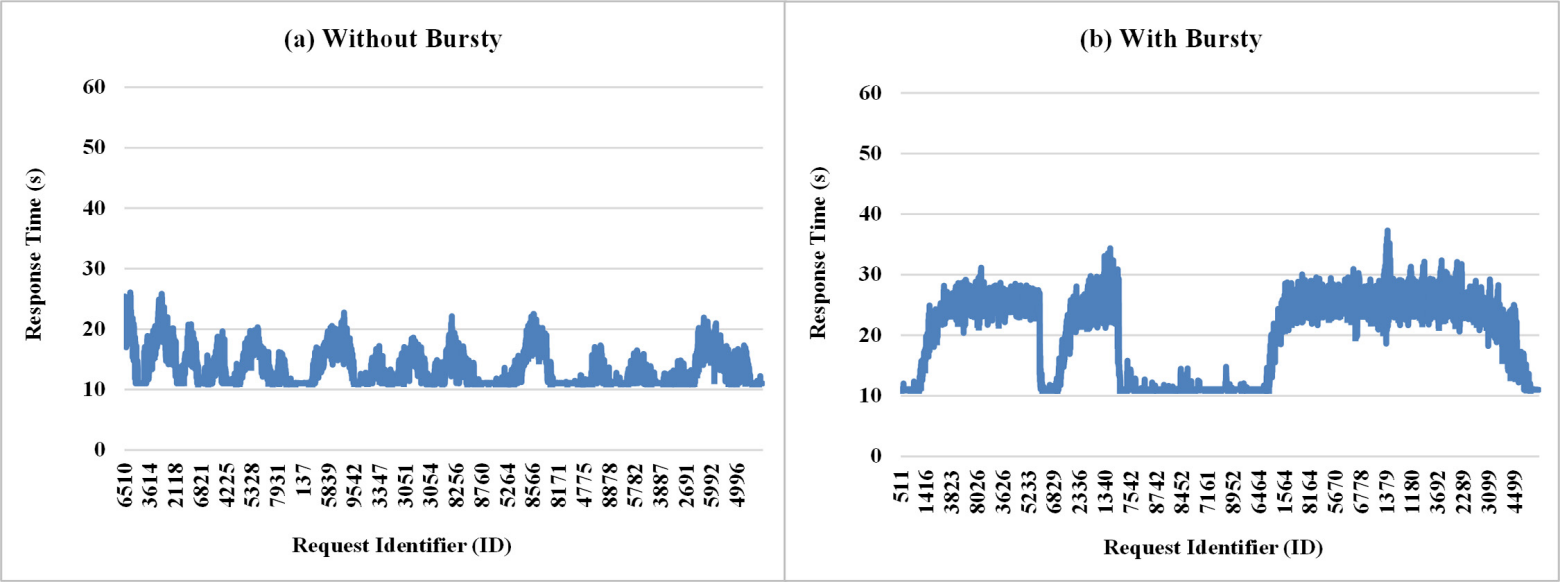
Samples of the response time of requests considering Bursty and Non-Bursty Workload with think time after response. Samples of the experiments where think times beginning after receiving the response from the previous request (a) without bursty, (b) with bursty.

The graphs in Fig 11 show the response times of requests sent to the private cloud, at runtime, observed during the execution of one run of each experiment considered in this subsection. This comparison shows how performance can be affected when the requests come in the form of bursts. According to the graph of Fig 11(B), there are moments when strong and irregular peaks at different intensity appear when burst conditions are created. This behavior does not occur with the same intensity when the arrival of process without bursts is considered, as can be seen in the graph shown in Fig 11(A).

**Fig 12:**
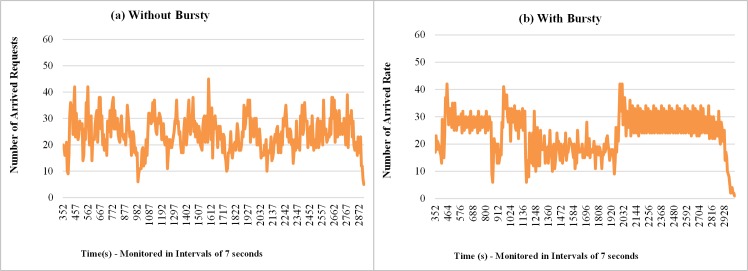
Samples of the number requests arrivals considering Bursty and Non-Bursty Workload with think time after response. Samples of the experiments where think times begin after receiving the response from the previous request (a) without bursty, (b) with bursty.

**Fig 13:**
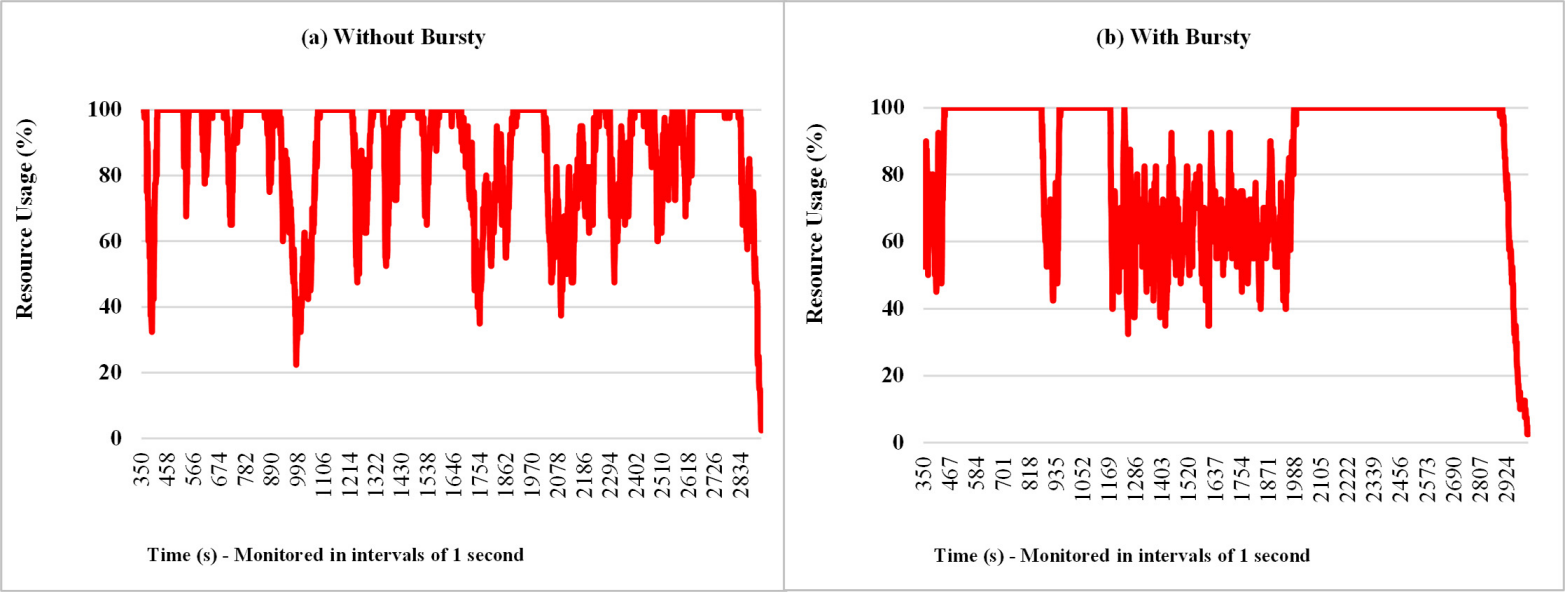
Samples of the resource usage percentage considering Bursty and Non-Bursty Workload with think time after response. Samples of the experiments where think times begin after receiving the response from the previous request (a) without bursty, (b) with bursty.

Figs 12 and 13 show samples of the amount of requests received by the broker and resource consumption percentage of the cloud, respectively. In both measurements (Figs 12(B) and 13(B)) when the requests arrive in the form of bursts, irregular peaks of different intensities appear, which leads to a greater variability in the results. For the results presented in the cases of workloads without bursts, it is possible to observe in Fig 13 (A) an overload of resource consumption, however, with lower intensity when compared with the case where the bursts are considered.

#### 7.2.2. Bursty and Non-Bursty Workload with Inter-Arrival Time after the Submission of the Previous Request

**Fig 14:**
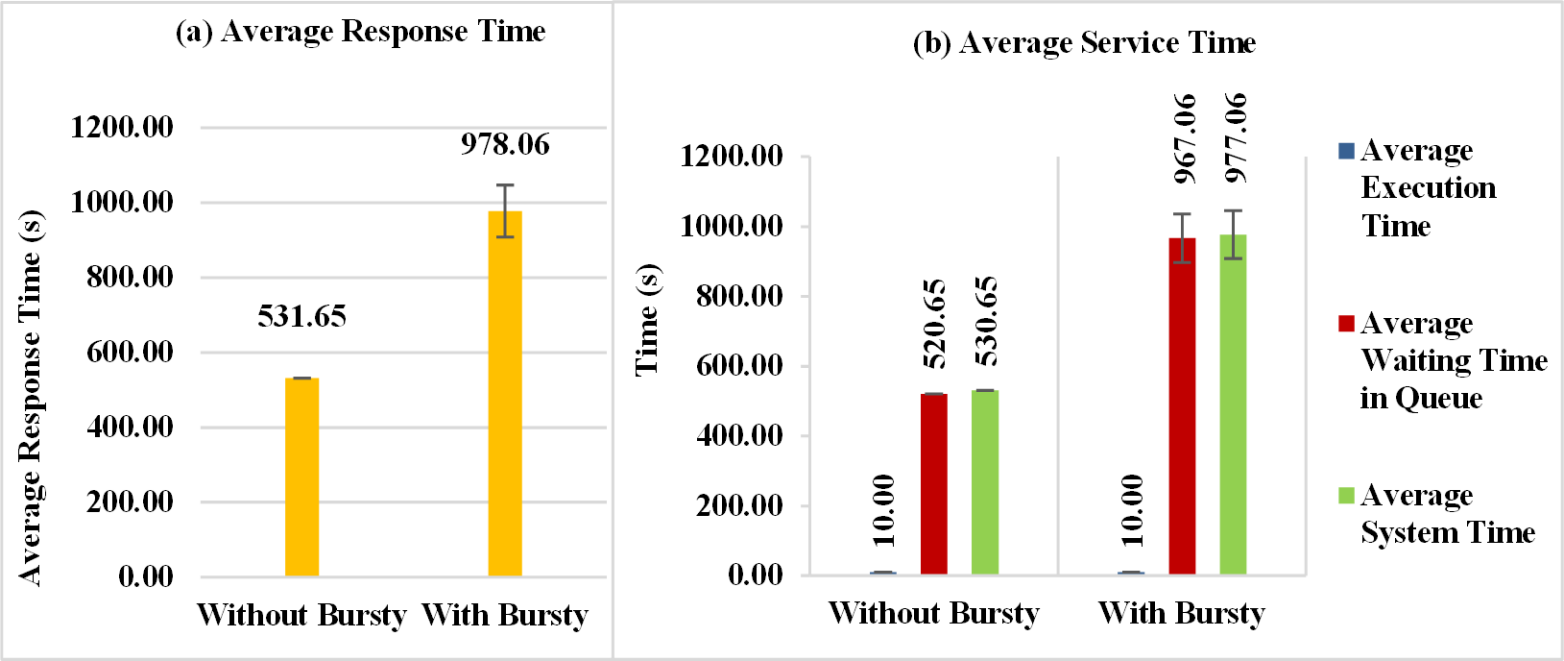
Response Variables considering Bursty and Non-Bursty Workload–think time after submission. Experiments that consider workloads with and without burst and that consider the beginning of the think time immediately after submission of the previous request (a) Average response time (b) Average service time.

In this section are presented the results of experiments that consider the time between two successive requests starts to count immediately after submission of the previous request. Similarly to the results discussed in the Section 7.2.1, when workloads with bursts are generated, the average response times become worse. In this case, as the requests arrive in a small period of time, in comparing with those considered in Fig 10, the degradation of the burst version is greater. Fig 14(A) shows an increase of approximately 84% in response time when workloads with bursts are considered. Even for situations with bursts in Fig 14(B), there is a significant increase in the average waiting time in the queue of the VMs to attend the requests. This most severe impact is related to the way the think times have been set, which need not to wait for the arrival of the response of requests to be initiated and the time between sending a request and the other becomes smaller.

**Fig 15:**
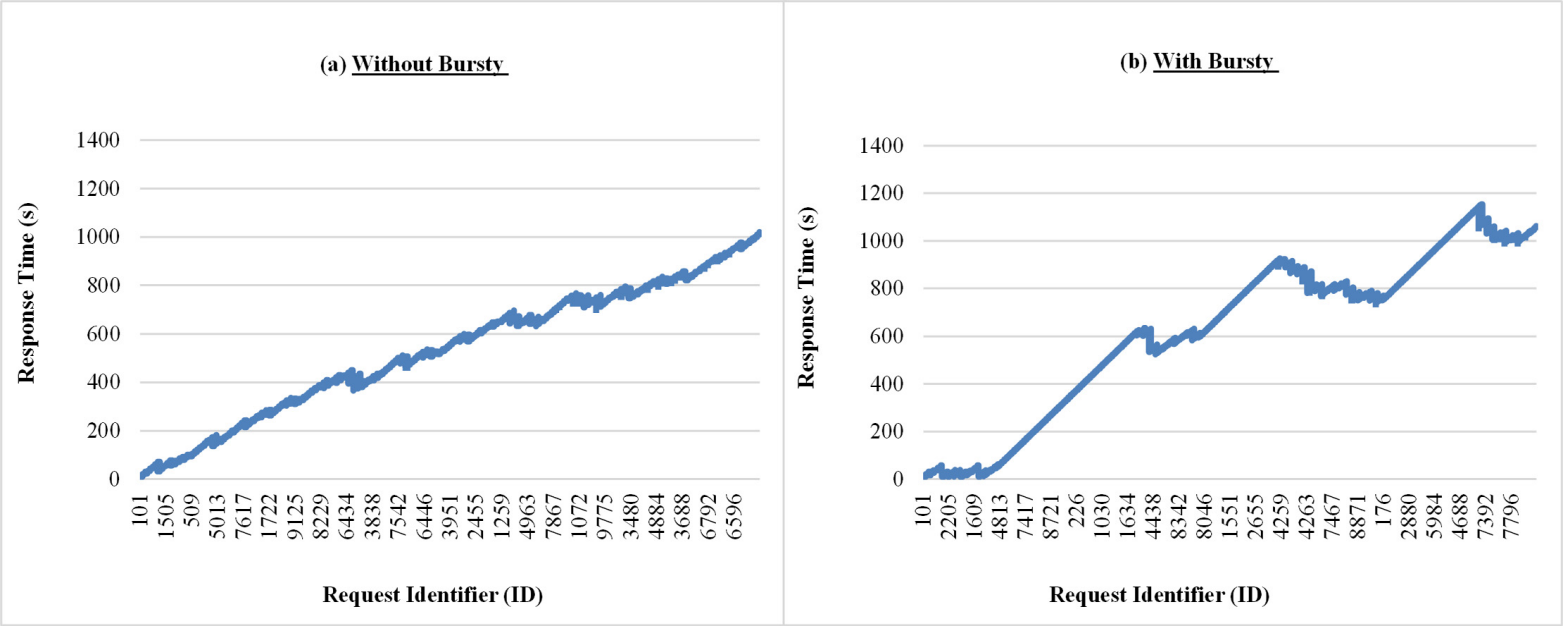
Samples of the requests response time considering Bursty and Non-Bursty Workload with think time after submission. Samples of the experiments that consider the beginning of the think time immediately after submission of the previous request (a) without bursty, (b) with bursty.

**Fig 16:**
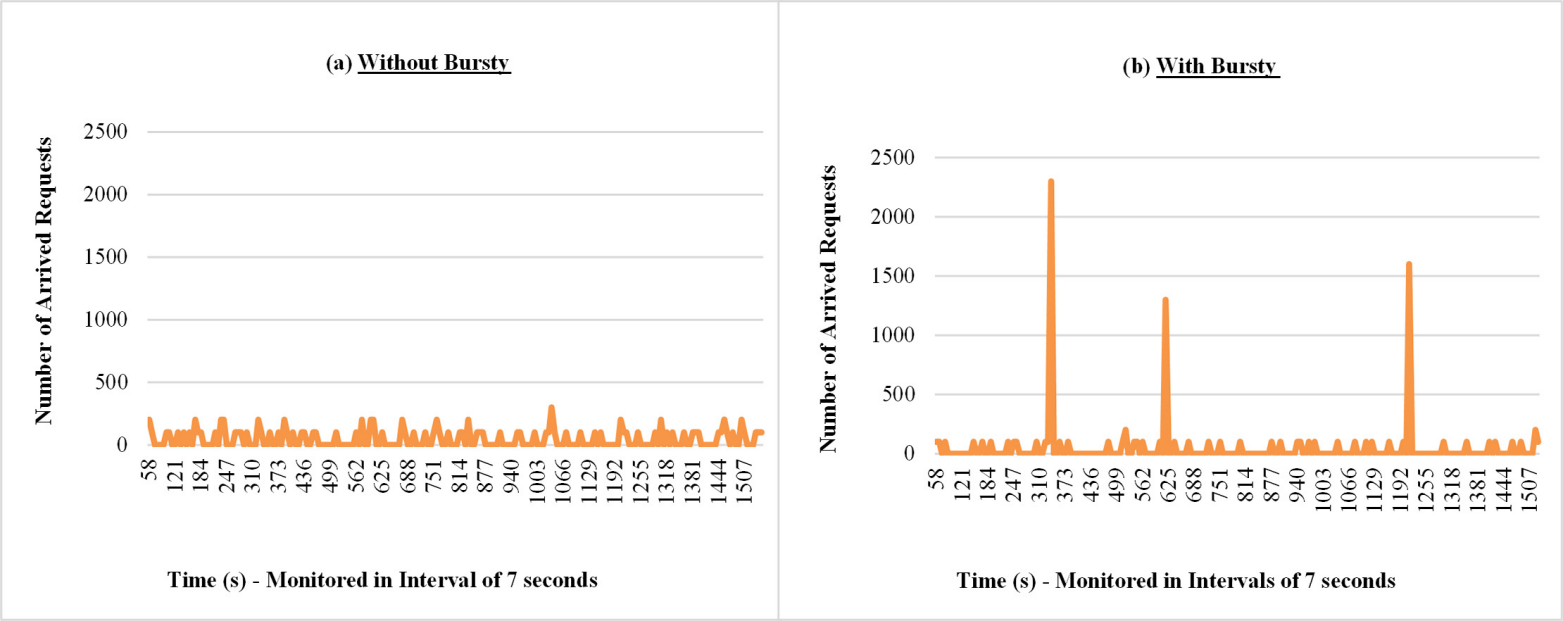
Samples of the requests arrivals number considering Bursty and Non-Bursty Workload with think time after submission. Samples of the experiments that consider the beginning of the think time immediately after submission of the previous request (a) without bursty, (b) with bursty.

**Fig 17:**
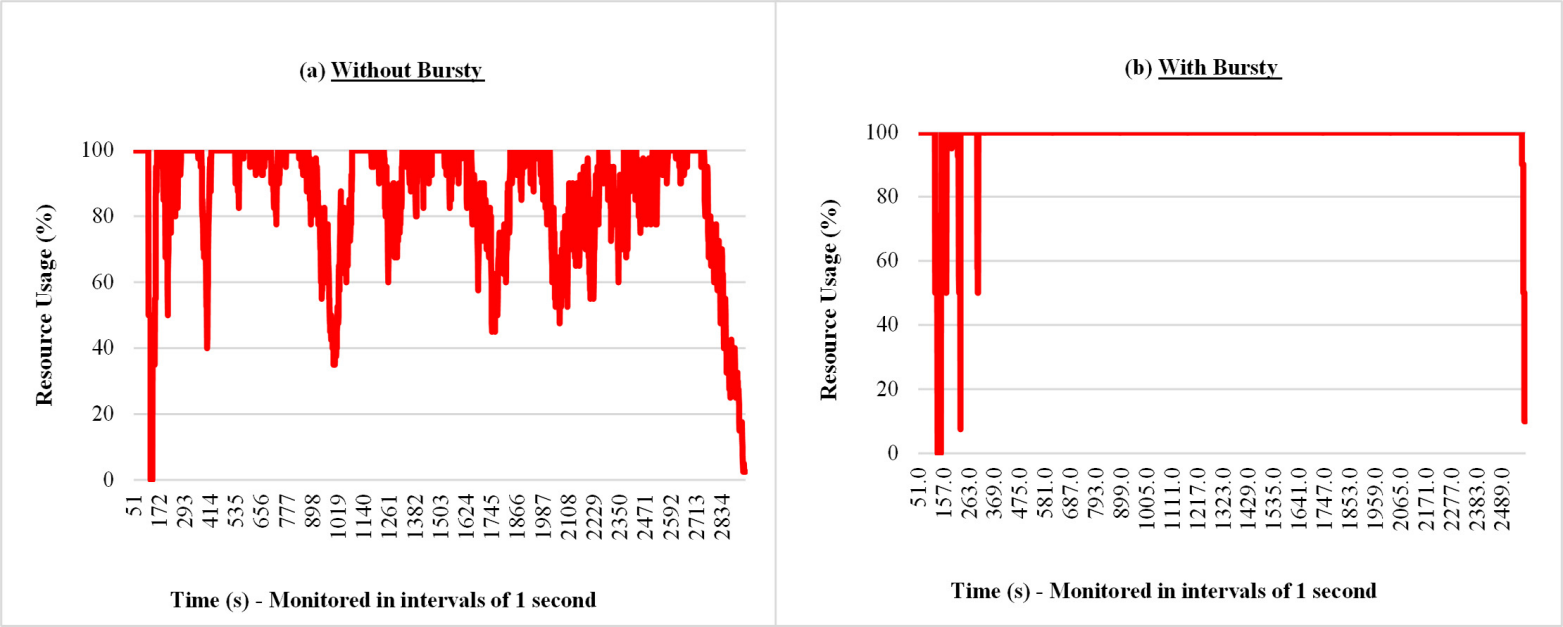
Samples of the resource percentage usage considering Bursty and Non-Bursty Workload with think time after submission. Samples of the experiments that consider the beginning of the think time immediately after submission of the previous request (a) without bursty, (b) with bursty.

The results in the graphs of Figs 15, 16 and 17 shows samples observed during a run of each experiment considered in this subsection, related to response times of requests, monitored number of requests at intervals of 7 secondsFig 15 shows, for situations with and without bursts, that the response times of requests increase over the time. This is because different models are used, although the averages of think time in both cases are the same (7 seconds). The interval between the arrivals of requests, on average, is smaller than the average running time of the requests, i.e., 10 seconds. This situation causes a backlog of tasks in the queues of the resources (VMs), hence a growing increase in waiting time in the queue that requests have to wait before being attended. According to Figs 16(B) and 17(B), when bursts are introduced in the arrival process of the requests, became more evident the existence of more severe peaks and variable intensities, both for the number of requests received and the percentage of resource consumption.

### 7.3. Network Latency

Simulation experiments considering the presence of network latency between the client and the Broker entities are provided in this section. In the experiments for the evaluation of the latency influence, the individual mode of submission with request vector was considered and two parameters were adopted for network latency. The first considers a homogeneous latency and is configured with a fixed value of 500 milliseconds. The second is heterogeneous and is generated by random values in the zero to 2000 milliseconds (ms) range.

**Fig 18:**
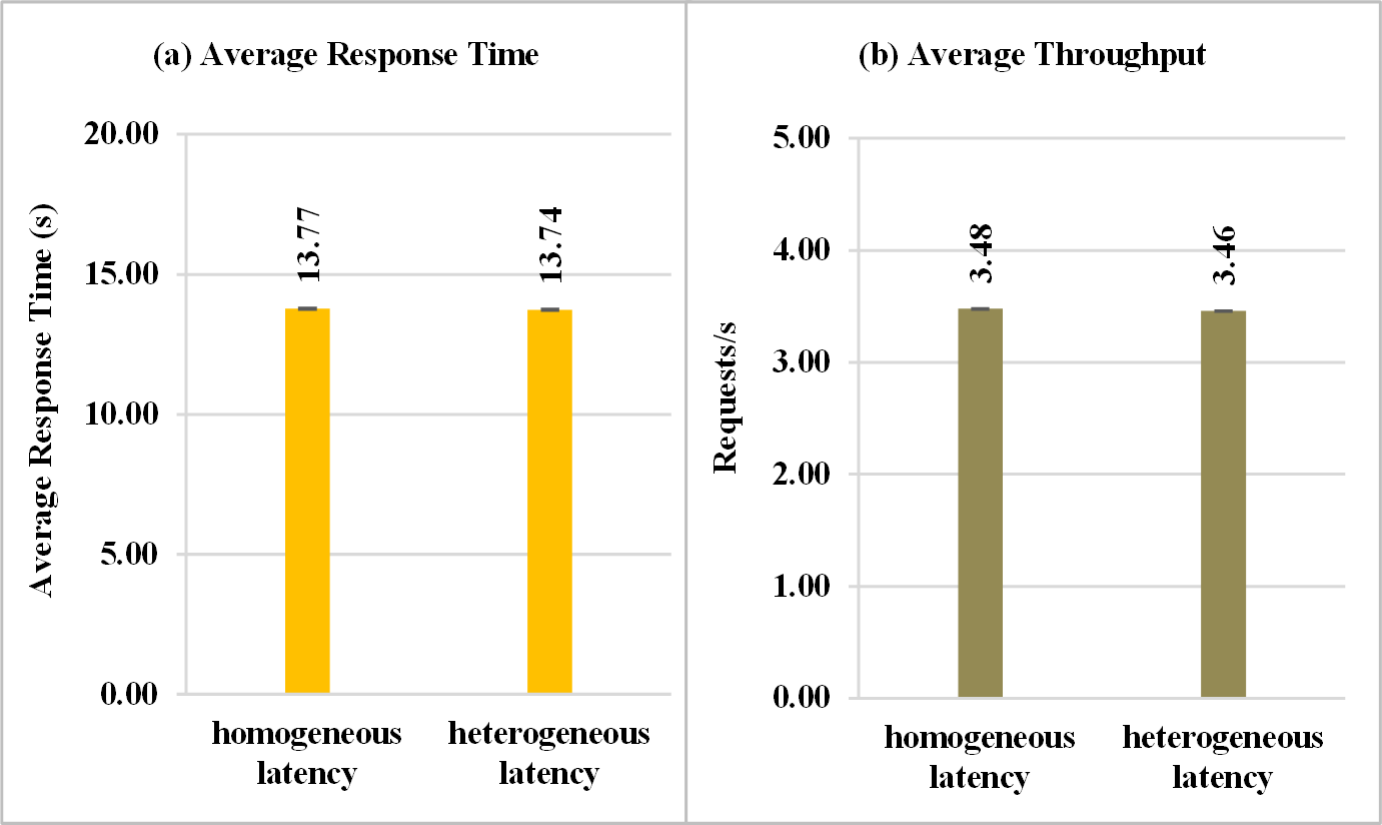
Response Variables considering homogeneous and heterogeneous network latency. Results of the experiments considering homogeneous and heterogeneous network latency between client and broker: (a) Average response time, (b) Average Throughput.

**Fig 19:**
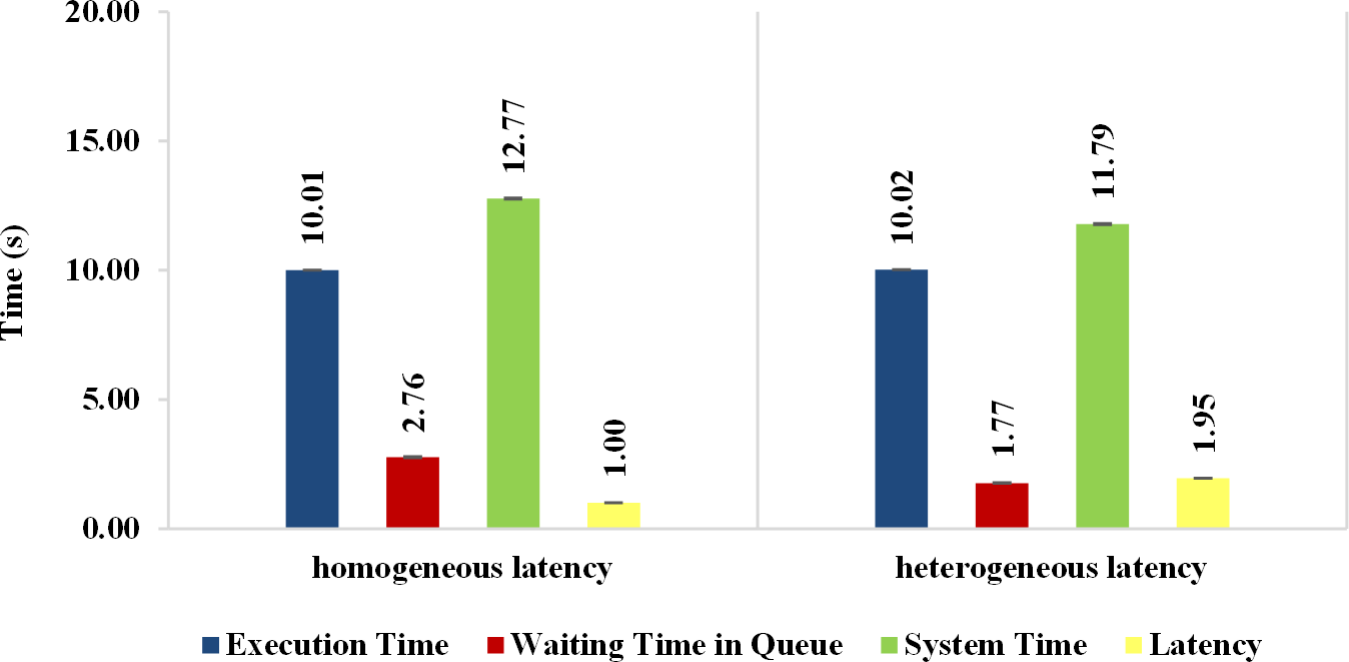
Average System Time and Average Latency for experiments that consider homogeneous and heterogeneous network latency. Results of the experiments considering homogeneous and heterogeneous network latency between client and broker.

Fig 18(A) shows that the average response time, when heterogeneous network latency is considered, promotes a slight improvement over the homogeneous latency. However, the throughput (Fig 18(B)) obtained was slightly lower for situations of heterogeneous latency, because the mean latency obtained for the heterogeneous approach is greater than the obtained for the homogeneous latency, as illustrated in Fig 19. Therefore, the average throughput is affected since successive requests in the heterogeneous case are submitted to the cloud with a slightly higher delay, softening the average waiting time in the queue and reducing the average response time. This behavior can be seen in Fig 19, where the results for the average system time, which is the sum of the average execution time and the average waiting time in the queue of the VM, as well as the average latency, are shown. The average running time on VMs is almost constant since the tasks have a homogeneous demand and the cloud resources are also homogeneous. On the other hand, for homogeneous network latency, the average waiting time in the queue showed an increase in comparison to the heterogeneous latency.

## 8. Conclusions

This paper addressed the simulation of cloud computing systems involving scenarios that consider the client representation.

A new client entity is proposed and it introduces new features to the CloudSim simulator, as it enables the creation of scenarios in which client behavior influences on the simulation and thus makes results more realistic. The entity is based on several important features that affect the performance of a cloud computing system. Among such features it is important to highlight: the existence of different ways of submitting individual or in group requests, time intervals between successive requests, and workload models that consider bursts in the request arrival process. The paper also describes a layered based simulation architecture, which was used as a basis to conduct a performance evaluation of the new simulation entity. The experimental results show the behavior of the client entity in the simulated environment and its influence on the performance of the services executed in a cloud.

The obtained results, considering the factors, levels and experiments used in this study show the importance of a client entity in a Cloud Computing Simulation. With the client layer it is possible to add greater detail to the scenario through intrinsic characteristics of the client, such as type of application executed, number of requests submitted by each client, submission mode of requests during the time of simulation and models of different workloads with or without the burst phenomenon in the arrival process. In addition to these basic features, much more can be added to the original Client class designed in order to increase the realism of the simulation, making the scenarios in the simulation closer to those in actual Cloud Computing.

Therefore, it is possible to conclude, based on results obtained from the experiments, that the new Client entity designed for the CloudSim simulation framework has a positive effect on the proposal of enhancing the simulation environment. It allows to the researchers several new insights into their simulation scenarios, approaching their evaluations and validations even more to a real world scenario of cloud computing systems.

Some studies have proposed increasingly complex simulation environments to validate new methodologies for broker intermediation [32, 33, 34, 35], predictive models considering QoS attributes [36], scheduling and allocation policies for virtual machines using different techniques such as ant colony [37], genetic algorithms [38], Particle Swarm Optimization (PSO) [39, 40, 41, 42] and other situations found in cloud computing systems.

It is very important for cloud computing simulators that all existing entities found in real cloud computing systems can be represented as they were proposed, validated and published in architectures such as the National Institute of Standards and Technology (NIST) cloud computing architecture [43].

Besides the contributions from the new propositions presented in this paper, additional suggestions for future researches are also presented as follows:

- Creation of clients that can connect to more than one broker, because in this way clients will not be limited to rely on just one intermediary entity, but on a set of them. This would avoid that clients have to be dependent on trading service requests with a single Broker;
- Implementation of clients that send heterogeneous requests, so that the characteristics of each request (size of input and output file, task length, execution mode, requested service) can be differentiated; this becomes important because in real world clients do not always request the same services by sending request after request. This is a way to make the client entity even more realistic and increase the complexity of the simulation, generating more effective results that could evaluate what was proposed via simulator in a more effective mode;
- Introduction of cost model in the clients, so that cost related parameters can be added to each request. A client would be characterized with "pay-as-you-go" requests and/or with monthly cost requests, for example;
- Incorporation of QoS advanced issues to clients, so that requests could be handled by considering several parameters of quality of service, not only the maximum response time, for example;
- Allow clients to "initialize" at any time and not just at the beginning of the simulation (in the first instants of the clock). This feature would also increase the realism imposed on simulation scenarios;
- Development of new modes of submission that include other types of characterization for the clients application. Thus, it is possible to cover a larger number of possible behavior performed by the clients related to the time they will send their requests to the broker.

As additional material of this work was included a supporting file named as S1 Table containing multiple spreadsheets in Excel format, which contains from the experiments plan to experimental results of samples containing data and information obtained through the CloudSim simulator output reports.

## Supporting Information

S1 Table Experiments and results data spreadsheets in MS-Excel format. A support information file is added to the set of publication files containing the data generated by simulation experiments with CloudSim.
(XLSX)
